# Identification of Novel Elements of the *Drosophila* Blisterome Sheds Light on Potential Pathological Mechanisms of Several Human Diseases

**DOI:** 10.1371/journal.pone.0101133

**Published:** 2014-06-26

**Authors:** Oleksii Bilousov, Alexey Koval, Amiran Keshelava, Vladimir L. Katanaev

**Affiliations:** Department of Pharmacology and Toxicology, Faculty of Biology and Medicine, University of Lausanne, Lausanne, Switzerland; Institut de Génomique Fonctionnelle, France

## Abstract

Main developmental programs are highly conserved among species of the animal kingdom. Improper execution of these programs often leads to progression of various diseases and disorders. Here we focused on *Drosophila* wing tissue morphogenesis, a fairly complex developmental program, one of the steps of which – apposition of the dorsal and ventral wing sheets during metamorphosis – is mediated by integrins. Disruption of this apposition leads to wing blistering which serves as an easily screenable phenotype for components regulating this process. By means of RNAi-silencing technique and the blister phenotype as readout, we identify numerous novel proteins potentially involved in wing sheet adhesion. Remarkably, our results reveal not only participants of the integrin-mediated machinery, but also components of other cellular processes, e.g. cell cycle, RNA splicing, and vesicular trafficking. With the use of bioinformatics tools, these data are assembled into a large blisterome network. Analysis of human orthologues of the *Drosophila* blisterome components shows that many disease-related genes may contribute to cell adhesion implementation, providing hints on possible mechanisms of these human pathologies.

## Introduction

*Drosophila* wing is a perfect model for diverse genetic analyses and is useful in different developmental studies, mostly due to existence of a wide range of mutations affecting wing development and relative simplicity of the wings tissues. *Drosophila* wing is composed of two epithelial layers which develop from a specific area of the wing imaginal disc, called the wing pouch, during metamorphosis. Upon its development, the wing tissue undergoes a series of well-described and strictly defined morphogenetic events [1]. After the wing pouch evaginates and folds along the midline, it passes through four key steps: apposition – when two basal surfaces of wing epithelia come together; adhesion – when junctions form between the apposed basal surfaces; expansion – when the wing blade expands as cells flatten; and separation – when basal surfaces separate from each other and a specific transalar apparatus differentiates. Each of these morphogenetic rearrangements happen twice: at prepupal and at pupal stages of *Drosophila* development [1].

Numerous studies have revealed the critical role played by integrins as the key mediators of the formation and maintenance of the developing *Drosophila* wing bilayer [2]–[4]. Integrins are transmembrane heterodimers formed by noncovalently associated α and β glycoprotein subunits with a large extracellular domain recognizing extracellular matrix (ECM) ligands and a short cytoplasmic tail binding to adaptor proteins. According to the Uniprot and FlyBase databases, five α- and two β-integrin subunits are encoded in the *Drosophila* genome. Among them only one βPS subunit (PS standing for “position specific”), encoded by *myospheroid* (*mys*) locus, and two α subunits: αPS1 – *multiple edematous wings* (*mew*) and αPS2 – *inflated* (*if*) have been shown to be required for the apposition of the dorsal and ventral epithelial sheets during wing morphogenesis. While the β-subunit is evenly distributed over most of the basal cell surface of wing discs, αPS1 and αPS2 subunits are exclusively expressed on the future dorsal and ventral wing epithelium, respectively [5]. Such position-specific allocation of integrin heterodimers of different composition is important for the subsequent accurate apposition and adhesion among the future intervein cells of evaginated wing pouch [6], where they form adhesion-like clusters called basal contact zones [1]. A defect in either integrin gene product can produce wing blisters – regions within the adult wing where the two surfaces are not apposed [4], [7]. Intriguingly, imbalanced amounts of αPS integrin subunits (e.g. by overexpression of any of them) leads to a similar dominant phenotype called Blistermaker phenotype [6], [8].

During wing development in *Drosophila*, integrins appear to provide a linchpin in the transalar apparatus that stretches from one wing surface to the other. The transalar apparatus is a mechanically continuous structure consisting of parallel arrays of microtubules and microfilaments anchored apically to the cuticle via hemi-desmosomes and basally to the opposite epithelial layer via the basal junctions [1]. Thus it is presumed that integrins mediate two distinct spatial and temporal functions during *Drosophila* wing morphogenesis: the mediation of cell-cell interactions by forming basal junctions and the cell-matrix interactions [1], [6], [8].

Despite wing tissue simplicity, its morphogenesis is a fairly complex developmental program and integrins are not the sole molecules involved in wing sheet apposition and adhesion. Many other proteins implicated in different signaling pathways (e.g. Wingless, Decapentaplegic, Notch, Hedgehog) also play important functions in wing morphogenesis and may contribute to wing blistering when functioning improperly. Processes regulating cell cycle, apoptosis, and epithelial-mesenchymal transition are also involved in wing morphogenesis and can regulate the dorso-ventral sheet apposition [9]. Vein/intervein formation is another example of a process which is far-standing from the integrin-dependent adhesion mechanics (as the vein cells do not express integrins and do not form transalar arrays [1]) but nevertheless may contribute to the wing blister phenotype when the cell fate determination shifts in favor of the vein cells which do not form connections with the opposite surface [10], [11].

This complexity should be considered when performing attempts to identify novel blisterome components – genes which upon mutation result in blister formation – and to ascribe such genes to the integrin-mediated adhesion. Several of such attempts have been previously performed using the FRT-FLP system inducing formation of somatic loss-of-function clones in the developing *Drosophila* wing [12], [13], disclosing several mutations causing the wing blister phenotype. However, these approaches, as well as sporadic descriptions of other blister-causing mutations, were far from exhaustive characterization of the *Drosophila* wing blisterome.

Here we use the UAS/GAL4 system to express the library of *Drosophila* RNAi lines [14] in the *Drosophila* wing. We randomly chose 1709 transgenic RNAi lines which target 1573 protein-coding genes or ∼11.3% of the total gene number in the release 5.51 of the *Drosophila* genome. The list contained genes defined by the gene ontology (GO) terms as involved in a wide range of biological processes, molecular functions, and cellular components and also included genes without any assigned GO terms (see Table S1). This analysis revealed a large number of genes previously never implicated in cell adhesion or blister formation, allowing identification of (a subset of) *Drosophila* blisterome. As we further show, human orthologues of many of these genes are implicated in a number of diseases, shedding light on the possible underlying mechanisms of these pathologies.

## Results

### Blister-causing genes uncovered by means of RNAi screening

**Figure 1:**
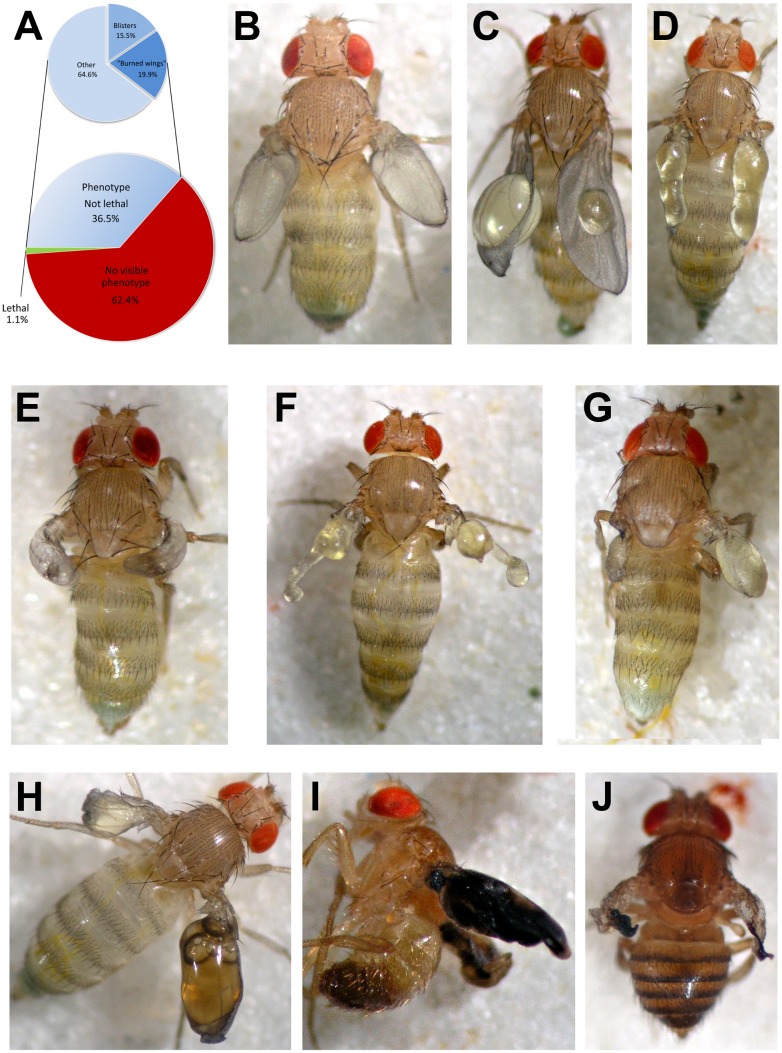
Results of wing-specific RNAi expression and the most typical manifestations of resulting blistered and “burned” wings. (**A**) General statistics of the RNAi screening. Blistered and “burned” wings were the most frequent phenotypes. (**B–G**) Examples of blistered wings with the blister occupying the whole wing (**B**, *RNAi-Fas1*) or the central position in the wing (**C**, *RNAi-rok*), or blisters accompanied with other wing defects such as narrow wings (**D**, *RNAi-CG10754*), “horned” wings (**E**, *RNAi-RnrL*), “swarovski” wings (**F**, *RNAi-Hsp83*), or “stump” wings (**G**, *RNAi-U2af50*). (**H–J**) Examples of “burned” wings. This phenotype may start as blistered wings in freshly eclosed flies (**H**) and with age develop into necrotic wings (**I**, *RNAi-Wg*), or reveal “burned” wings throughout the adult life (**J**, *RNAi-mys*).

The RNAi lines were crossed with the *MS1096-Gal4* driver line – an effective driver-construct for wing blister production together with appropriate UAS-constructs [9], [15]. This transgene directs strong GAL4 expression in the dorsal part and weaker in the ventral part of the developing larval and pupal wing [16]. The resulting *MS1096-Gal4; UAS-RNAi* flies were analyzed and the parental RNAi transgenes grouped depending on the observed wing phenotype. Almost two thirds (62.4%) of the analyzed RNAi lines gave no phenotype, 1.1% were lethal, and visible phenotypes (excluding lethality, but including semi-lethality) were scored for 36.5% of the analyzed RNAi lines (Fig. 1A).

Among the phenotypes observed, the “blister” and the “burned wings” phenotypes were the most frequent (Fig. 1A). A wing blister is a bubble in a wing often filled with hemolymph (Fig. 1). Its appearance correlates with disruption of integrin-mediated cell adhesion between the dorsal and ventral epithelial sheets of a wing [17]. This general phenotype can be split into smaller sets depending on whether the blister occupies the whole wing or only parts of it, and also on the accompanying other defects of wing formation (Fig. 1B–G). The “blister” group is represented by RNAi lines targeting 91 genes (5.7% of the screened lines, see Table S2). Among the affected genes are *Delta*, *blistery*, and *inflated*, previously identified as blister-causing in somatic clone analysis [8], [12]. Delta is a ligand activating the developmentally important Notch signaling cascade [18], the *blistery* protein product (human orthologue – tensin) has an actin binding function and acts as an adaptor stabilizing integrin adhesive contacts in *Drosophila* [19], while *inflated* encodes the αPS2 integrin subunit [3], [8]. Another overlap with previously published data is *parvin*, implicated in the integrin adhesion in *Drosophila* [20] and mammals [21]. However, the majority of the genes (see Table S2) have never been previously implicated in wing blistering.

The “burned wings” phenotype with warped and dusky wings also comes in different manifestations (Fig. 1H–J) and was represented by 120 genes (7.3% of the screened lines, see Table S3). Importantly, 39 lines developed “burned wings” upon ageing of blistered wings of newly eclosed individuals (Fig. 1H, I). We assumed that even with the RNAi lines which produce “burned wings” at birth, wings of the earlier pupal stages contained blisters. In agreement with this, we found that the RNAi targeting *myospheroid* which encodes the βPS integrin previously found to produce blisters in somatic clone analysis [8] gave rise to the “burned wings” phenotype (Fig. 1J). Some other examples of overlaps of genes within the “burned wings” category with previously described blister-causing mutations were found (see Table S3). Further evidence in favor of inherent similarity of the “blister” and “burned wings” phenotypes comes from the observation regarding three genes of our analysis list (CG8440, CG9193, and CG9998) which were covered with two RNAi lines each, of which one was producing the “blister”, and the other – the “burned wings” phenotype.

### Construction of the *Drosophila* blisterome. Identification of the mainstream functional modules and pathways

**Figure 2:**
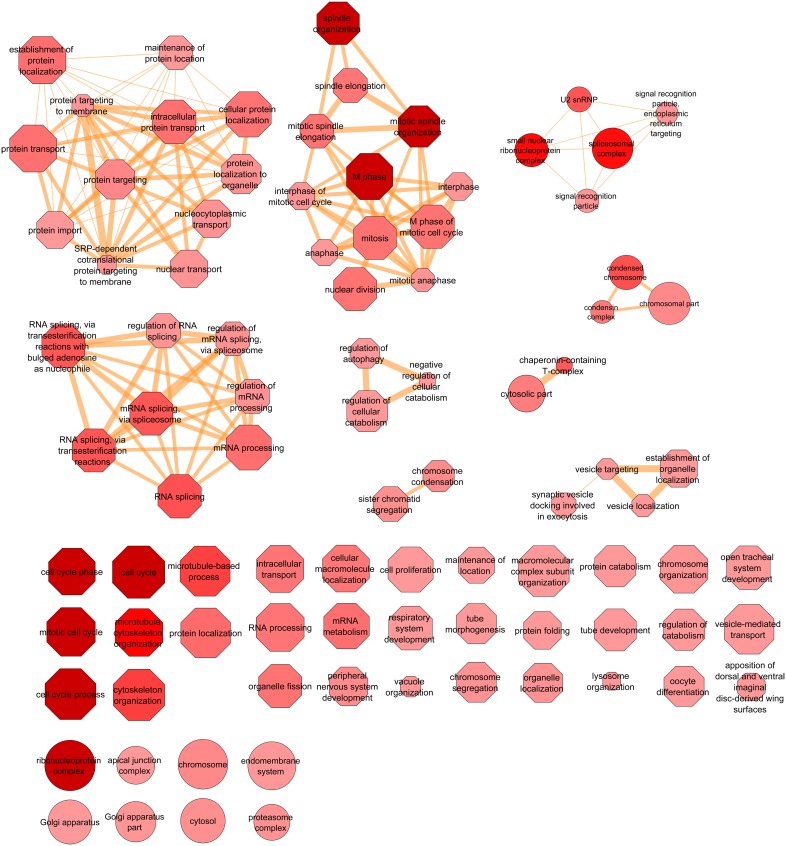
Semantic clustering of the GO terms enriched in the blister-causing group, compared to the annotations of all screened genes. Enriched GO terms are shown as nodes (“biological processes” as octagons and “cellular components” as circles), and the top 3% of the strongest GO term pairwise similarities are designated as edges in the graph. The node radius relates to the generality of the terms, where smaller nodes imply more specific terms; the supplied p-values/enrichments are shown using color shading, where more saturated color of the node implies more over-represented GO terms. The semantic similarity between two GO terms is shown by the thickness of the edges, where thicker edge implies more semantically similar GO terms connected by this edge.

We united the “blister” and “burned wings” phenotypic categories into a single one resulting in 208 genes supposedly regulating, one way or another, adhesion of the two wing epithelial layers. This group of genes was supported by 221 RNAi lines (13 and 2 genes were targeted by 2 and 3 RNAi lines respectively). *In silico* GO classification analysis by the DAVID bioinformatics tool revealed that this set of genes is enriched with 86, 20, and 8 terms from “biological process”, “cellular component” and “molecular function” categories respectively (see Table S4). We applied the semantic similarity measure to cluster the over-represented GO terms. This analysis identified the major groups of GO terms of genes involved in wing blister formation (Fig. 2). Surprisingly, in addition to the expected groups (such as apposition of the wing surfaces), the major over-represented groups of GO terms were related to protein transport, cell cycle, mRNA splicing, catabolism, vesicular trafficking, and others (Fig. 2).

To saturate our output list, we further screened through the available published data for genes which upon improper functioning led to wing blistering. We found overall 168 genes disclosed in 109 publications (see Table S5) among which 123 caused this phenotype due to their loss-of-function, and 45– because of their gain-of function. Out of them, 63 genes were screened in our analysis, and 18 (or 29%) were detected as the blister-causing (see Tables S2 and S3). This ratio of overlap is low as compared to the expected false-negative rate of our screen, which we assumed to be similar to that previously obtained in the genome-wide RNAi screen of Notch signaling components (29%) [14] as we used the same library of RNAi lines (the false-positive rate in the Notch screening was estimated as 7%). We note that the 168 genes we assembled from the published literature originate from different experimental approaches which may have varying false-positive values. In an attempt to overcome this complication, we combined the two datasets, resulting in the list of 358 blister-causing genes, and performed network analysis with it. We used the NetworkAnalyzer tool and the BioGRID interaction database and found that of the 358 genes, 151 cluster together resulting in a network with the average number of neighbors = 2.147, a two-fold improvement over the network properties of the list of genes originating from our screening only (see Fig. S1A, B).

The list of protein-protein interactions available for *Drosophila* is far from saturation and is less complete than e.g. in the case of human proteins. *Drosophila* has been widely used to model human pathologies, as some 75% of human disease-related genes have orthologues in the fly; the overall sequence identity between the orthologues is about 40% but can reach 80–90% within the conserved functional domains [22]–[24]. For the 358 candidate *Drosophila* blisterome genes we found 877 human orthologues; 41 *Drosophila* genes of this list did not reveal human counterparts (see Table S6). We next re-analyzed the network properties of the 358 *Drosophila* genes, adding the BioGRID-listed interactions of their identified human orthologues. The resulting network includes overall 292 connected components out of which 288 form a single large subgraph, with the average number of neighbors = 6.37 (see Fig. S1C).

Genes related to a given phenotype or physiologic/pathologic process appear tightly connected within an interaction network in contrast to unrelated ones, as has been shown e.g. for toxicity modulation in *S. cerevisiae* [25] or cancer progression in humans [26], [27]. We argued that, reciprocally, genes which are densely connected within a network have a high probability to produce the same phenotype and hence to be involved in the same biological process and/or developmental program. This notion is supported by comparison of our result to Monte Carlo simulations of 100 networks built by the same algorithm for 358 randomly selected *Drosophila* genes. For each network we evaluated 3 criteria characterizing its connectivity: the clustering coefficient, the number of connected components, and the average number of neighbors per node. The respective values for the *Drosophila* blisterome (see Fig. S1C) were ca. 2.5, 11 and 3.7 times higher than medians calculated for the Monte Carlo simulations set (Fig. S2, *p*<0.0001) thus proving a greater degree of cooperation among the components of our network. We thus argue that our screening for the *Drosophila* blisterome components, supplemented with an exhaustive literature screening and bioinformatics analysis, resulted in identification of a significant portion of the *Drosophila* blisterome network.

**Figure 3:**
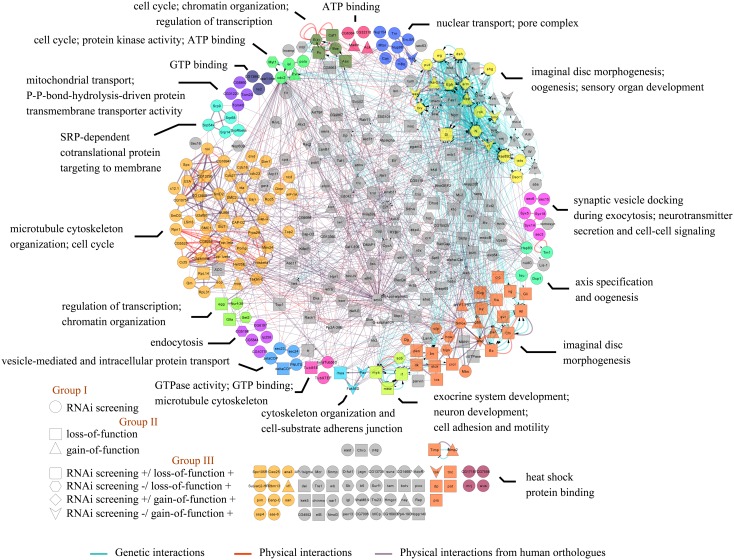
The *Drosophila* blisterome. Each gene is an independent node, with edges between them being interactions of the genetic (cyan) or physical (red) nature, or being inferred from the physical interactions among their human orthologues (lilac). Nodes are color-coded and grouped into functional clusters according to their annotation terms; interactions within each functional cluster are shown by bold edges. Grey nodes represent genes which failed to be clustered. Genes not interconnected into the large blisterome network are grouped below it; however, some of them are color-coded, because they still belong to functional clusters of the major network. Nodes are given as circles if coming from our RNAi screening only, rectangles or triangles if coming solely from previous loss- or gain-of-function analysis, or other symbols if coming from both our and previous analysis; complete description of the node shape coding is given in the lower left corner of the Figure.

Application of the DAVID gene functional classification tool has revealed 19 functional modules within the *Drosophila* blisterome as shown in Fig. 3. Genes involved in cell adhesion, cytoskeleton organization, cell cycle, regulation of transcription, mRNA splicing and processing, protein transport, vesicular trafficking, and imaginal disc morphogenesis appeared to be the prevailing components of the *Drosophila* blisterome. We next analyzed which pathways the identified proteins belonged to using the KEGG database. This assigned 112 proteins (31% of the total list) to different pathways, of which the Spliceosome, TGFβ, and Proteasome pathways were statistically enriched over the initial list of screened genes; other pathways present were e.g. the Progesteron, Wnt, or Endocytosis pathways (see Table S7).

### Components of the *Drosophila* blisterome are orthologous to many human disease-related genes which are tightly interconnected

**Figure 4:**
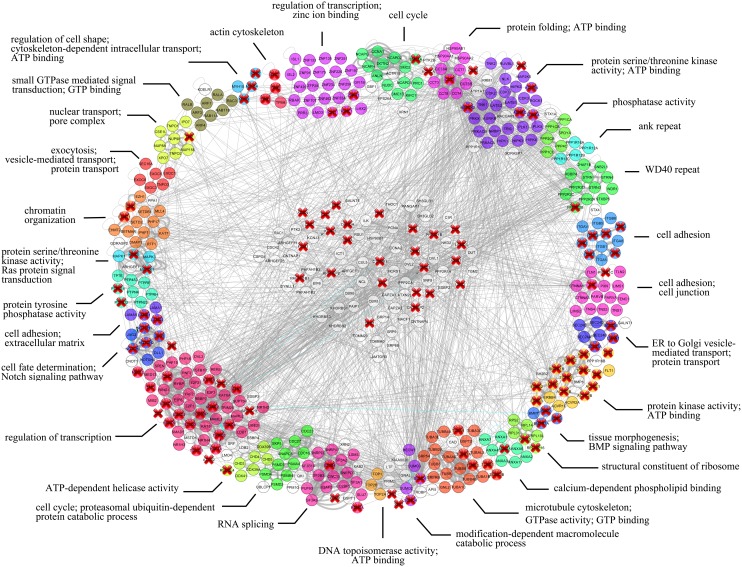
The network composed of human orthologues of the *Drosophila* blisterome components. Physical interactions are shown in grey, phenotypic – in blue. Disease-related genes are marked by red crosses. Nodes are color-coded and grouped into separate functional clusters according to their annotation terms; interactions within each functional cluster are shown by bold edges. Grey nodes represent genes which are not enriched by any term. However, some of these nodes were placed close to existing clusters if most of their connections were with its members; the remaining nodes with promiscuous interactions were grouped in the center of the network. To reduce Figure complexity isolated nodes were removed leaving only the highly interconnected ones.

Based on the orthology prediction approach we next “translated” the here-identified *Drosophila* blisterome into the human orthologous network. Among the total 877 found orthologues, 468 produced a large subgraph containing highly interconnected nodes with 31 functional clusters similar to those of the *Drosophila* blisterome (Fig. 4 and see Table S6). The rest 409 human counterparts appeared to be represented as isolated nodes with no reported interactions either with the major subgraph or among themselves and were discarded from subsequent analyses as the likely false-positives.

**Figure 5:**
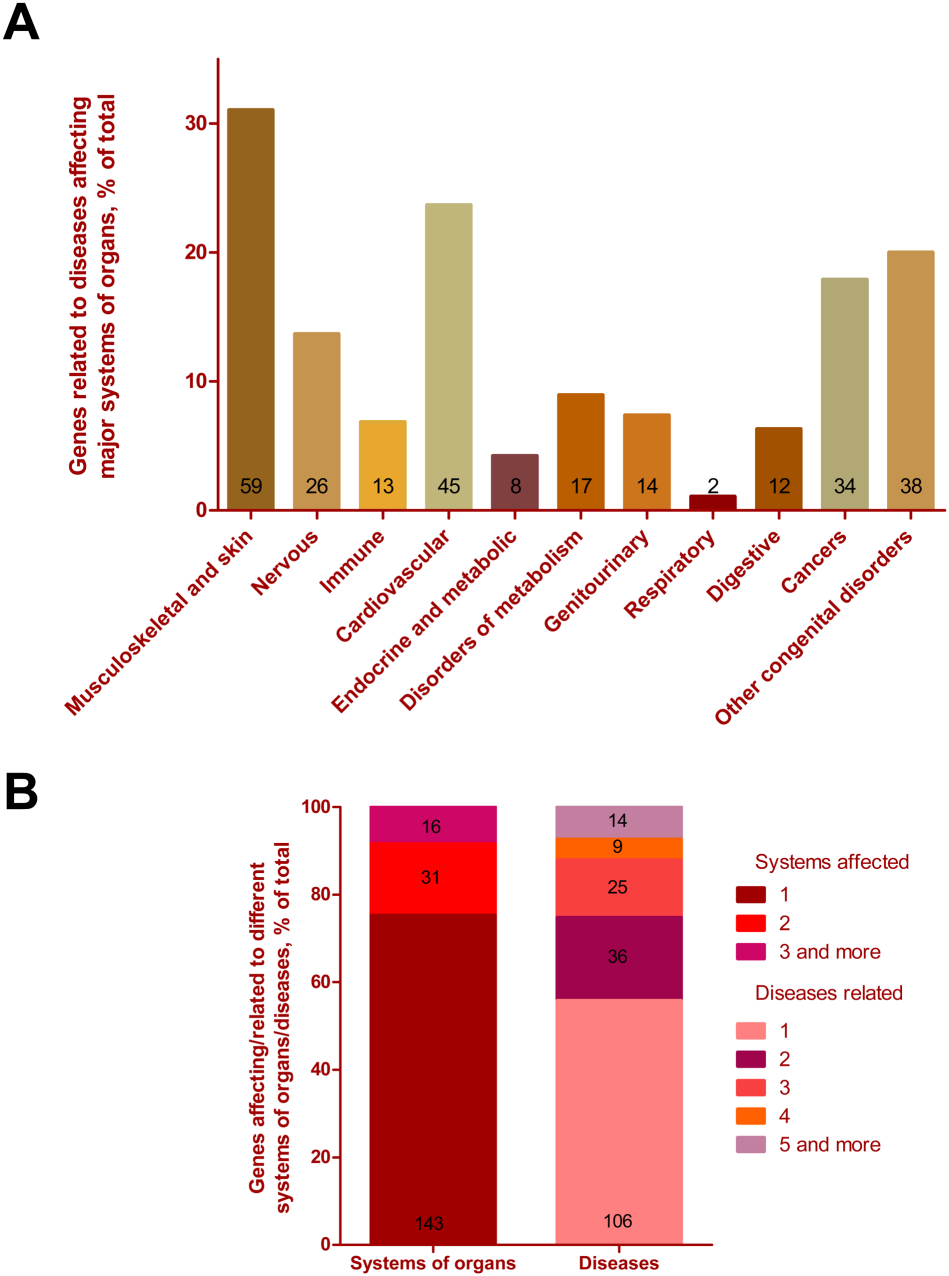
Human blisterome orthologues linked to diseases and disorders. (**A**) Distribution of the disease-related genes among major systems of organs affected by specific maladies associated with these genes. (**B**) Distribution of the disease-related genes by the number of systems they affect (left bar) (see Table S9) and diseases they cause (right bar) (see Table S8). The quantity of assigned genes is indicated by numbers on the graphs on both panels (A) and (B).

Disruption of the cell-cell contacts (e.g. desmosomes) in humans can lead to diverse skin and other connective tissues pathologies, including epidermolysis bullosa [28], [29]. Mutations of the genes for integrins or other structural components required for cell adhesion may lead to blistering in the skin and oral cavity [30]–[32], similar to the blister phenotype we observe in the *Drosophila* wing. Thereby we questioned whether the novel blisterome components we identified here have disease-related orthologues in humans. Among the total 877 found human orthologues, 190 genes are disease-related or contribute to susceptibility to multifactorial disorders – at least 260 diseases or disorders in total (see Table S8). Interestingly, 120 (two thirds) of those disease-related genes belong to the highly interconnected subgraph shown on Fig. 4. However, we grouped the whole number of disease-related genes according to the systems of organs where pathologies are manifested (Fig. 5 and see Table S9). After this grouping 11 categories emerge with the three most over-represented being: the musculoskeletal apparatus and skin –31%, cardiovascular –23.7%, and cancers –17.9% (Fig. 5 and see Table S9). Importantly, this list includes not only diseases of the connective tissue such as epidermolysis bullosa, xeroderma pigmentosum or skeletal dysplasia, but also other maladies such as retinitis pigmentosa, cardio- and myo-pathies, blood coagulation defects, primary immunodeficiency, diabetes, different cancers, etc. (see Table S8). Our results suggest that many diseases may be linked to various aberrant processes (e.g. mRNA splicing, protein folding, vesicle trafficking, etc.) which may be parts of general developmental programs alteration of which eventually may lead to cell adhesion impairment as one of the potential mechanisms of these human pathologies.

## Discussion

*Drosophila* wing morphogenesis is a highly complex developmental program, with many biological processes standing behind it. Successful formation of a mature adult fly wing depends on their harmonious interplay and cooperation. Wing blistering is a clearly visible phenotype revealing an impairment of specific wing tissue morphogenetic events, consequently resulting in aberrant adhesion of the two opposite wing epithelial sheets. Since the time when integrins had drawn attention of scientists to their role in wing morphogenesis and their functions had been implicated in the apposition of the dorsal and ventral wing epithelial layers [4], [6], [8], [33], to our knowledge, only two studies were done in order to find other participants involved in this process [12], [13]. Although genes resulting from these analyses have been attributed to integrin-mediated adhesion, it is clear that cellular processes may affect, sometimes indirectly, cell adhesion between the basal surfaces of the developing epithelial layers.

General architectural organization of the integrin-mediated cell adhesion machinery in *Drosophila* wings may be represented as a structure, which occupies three main “floors”. At the “basement” there lies the extracellular matrix, where adhesion-associated components like metalloproteinases, their inhibitors [34] and various integrin ligands [35]–[41] are localized. The “first floor” is at the the plasma membrane and is occupied by integrins themselves and by other adhesion receptors and their supporting molecules [42], [43], like the heparane sulfate proteoglycan syndecan [35]. The “second floor” is filled by a variety of inter-connecting adaptor proteins, like Pinch (encoded by *steamer duck*) [44], parvin [20], integrin-linked kinase [45], talin (*rhea*) [46], tensin (*blistery*) [19], [47], short stop [48], Wech [49], etc., which link integrins to the “third floor” – actin cytoskeleton and/or microtubules, which are necessary for the formation of transalar apparatus in the developing *Drosophila* wing.

Independently from the cell adhesion function of the integrin-mediated machinery, all the “floors” are governed by multiple functional molecular switches: serine/threonine, tyrosine kinases and phosphatases [42], [43], Rho family of GTPases [50], glycosyltransferases [51], etc., which proceed or terminate cell adhesion processes. There is also a “service staff”, like Rab11 which is involved in trafficking of the βPS integrin [15], or the ecdysone regulatory pathway and the bHLH protein Delilah (encoded by *taxi*), which control integrin expression [52], [53]. It has been also shown that alternative splicing of integrins alters interaction with their ligands [54], [55]. Loss of the receptor-ligand specificity, resulting from improper mRNA splicing, may produce improper formation of the basal contact zones between the opposite surfaces and as a consequence – wing blisters. This may be a possible explanation why one of the over-represented GO terms of *Drosophila* blisterome components is mRNA splicing (Fig. 2 and see Table S4).

At the pupal period of *Drosophila* metamorphosis enormous proliferation of rough endoplasmic reticulum is observed in wing epithelial cells during the basal adhesion stage. Numerous basally located Golgi bodies in addition to the usual apical Golgi are present. These ultrastructural features are characteristic of systems displaying rapid constitutive exocytosis [1]. As we have shown (Fig. 3), one of the functional modules of *Drosophila* blisterome is a vesicle-mediated trafficking and protein transport. According to the phenotypes we observed, downregulation of the genes from this module also results in wing blister appearance.

Following the final “larval” mitosis that occurs shortly after pupariation, most cells are in the G2 arrest for the remainder of the prepupal period. Then the final mitosis of wing development occurs between 15 and 24 hours with the mitotic peak at 17–18 hours [56]. Our results suggest that impairment of the cell cycle (Fig. 3) during these stages may also affect apposition and adhesion of epithelial layers and result in wing blister formation.

Finally, alignment of the two epithelial layers of developing wing is a very subtle process. Any manipulation that causes regional disparities in size of dorsal and ventral wing surfaces - e.g. altered growth, apoptosis, and disruptions in patterning - can lead to a misalignment where cells in one surface are unable to partner with cells in the other surface, which can lead to blistering. Because the *MS1096-Gal4* driver drives stronger RNAi transgene expression in the dorsal part and weaker in the ventral part of the developing larval and pupal wing [16], it may produce such dorsal-ventral mismatches.

In summary, we find 190 novel genes involved in apposition and adhesion of the two *Drosophila* wing epithelial layers; the precise function of these genes can now be studied with the full range of *Drosophila* genetics methods. Second, we have compiled the scattered data on blister-causing *Drosophila* mutations and combined these data with our findings to produce the first estimation (likely incomplete) of the *Drosophila* blisterome. Its analysis surprisingly reveals that not only established cell adhesion components, but also components ascribed to such cellular processes as cell cycle or mRNA processing constitute important clusters within the blisterome. Third, we have “translated” this *Drosophila* blisterome into the human network, revealing many components which may cooperate together in general developmental programs. And fourth, we predict that several human diseases may have aberrant adhesion as a potential underlying molecular feature inducing pathogenesis. If true, new treatment strategies for such diseases may be envisioned in future.

## Materials and Methods

### Fly crosses and microscopy

The following *Drosophila* lines were used: *MS1096-Gal4* (Bloomington *Drosophila* Stock Center, USA) and the RNAi lines targeting chosen genes (see Table S1), obtained from the Vienna *Drosophila* RNAi Center library, Austria [14]. All crosses were performed on standard media at 25°C. Whole flies were photographed through a Carl Zeiss Stemi 2000 binocular using the Olympus CAMEDIA C-5060 Wide Zoom.

### Analysis of gene lists using DAVID bioinformatics resources

We applied the DAVID gene functional classification tool [57] to, first, uncover the functions of the blisterome components and, second, to cluster them into functional modules accordingly. This tool uses a set of techniques which enable to classify input genes into functionally related groups on the basis of their annotation term co-occurrence. Such a way of clusterization of the *Drosophila* blisterome members appears more justified than grouping them e.g. by the Markov Clustering algorithm (MCL) which creates transition matrices by a random walk through the graph in order to discover where the flow tends to gather, and therefore, where clusters are [58]. For more precise clustering MCL-like algorithms frequently use edge weights, but BioGRID does not dispose a universal reliability score system of interactions since they are obtained from various sources each using its specific scoring system or none at all. Further, the *Drosophila* blisterome we characterize here is incomplete with many nodes and edges missing, thus utilization of the MCL-like algorithms would likely lead to artifact clustering, unlike the DAVID functional clustering tool.

To use the DAVID tool, the gene lists (see Tables S2, S3) were checked for enrichment of associated Gene Ontology (GO) terms, separately for each of the three GO categories (biological process, molecular function, and cellular compartment); GO annotations of the whole screened 1573 genes (see Table S1) were used as the background. To examine the significance of gene-term enrichment with a modified Fisher’s exact test, the *p*-value cutoff was set at <0.05. To globally correct enrichment *p*-values to control family-wide false discovery rate, Benjamini testing correction technique was applied in Tables S4 and S7.

To bypass the GO terms redundancy (e.g., when the terms being analyzed are in a parent-child or siblings relationship) and to cluster them with subsequent emission of only significantly enriched cluster representative terms (the choice was guided by the input *p*-values, previously corrected by the Benjamini correction technique), we applied the Resnik’s semantic similarity measure based on the node-based and MICA (most informative common ancestor) approaches. For description of this measure, which is believed to be the most appropriate and reliable for most biological studies, as well as for a review of other semantic similarity measure techniques see [59]. Here we used the REVIGO (Reduce + Visualize Gene Ontology) web-tool (http://revigo.irb.hr/) with a set cutoff value *C* = 0.5 (one of the semantic similarity values pre-defined by this tool), which corresponds to the “small” list of GO terms in the outcome. The REVIGO’s algorithm and its resulting values are described [60]. To obtain high resolution images the “interactive graphs” built after the REVIGO analysis were imported into the Cytoscape software [61].

### Orthology prediction, search for disease-related orthologues and “translation” of the human orthologues’ interactions from the *Drosophila* network

Human orthologues for *Drosophila* genes were found using several databases: OrthoDB (http://cegg.unige.ch/orthodb6) [62], InParanoid (http://inparanoid.sbc.su.se/cgi-bin/index.cgi) [63], FlyBase (http://flybase.org/) [64], and KEGG (Kyoto Encyclopedia of Genes and Genomes, http://www.kegg.jp/) [65]. The data were downloaded and unified by an in-house-made PERL scripts using Flybase (http://www.flybase.org) and the BioMart interface of the Ensembl project (http://www.ensembl.org/index.html) for ID conversion. Subsequently the data were manually verified and extended by information from the GeneCards (http://www.genecards.org) [66] database (see Table S6).

The human orthologues of blister-causing genes were analyzed for related diseases using the OMIM (Online Mendelian Inheritance in Man catalog, http://www.omim.org/) [67] and KEGG databases and information was automatically extracted and compiled by in-house PERL scripts. ICD-10 (International Classification of Diseases) classifications (http://apps.who.int/classifications/icd10/browse/2010/en) were added manually (see Table S8).

To “translate” the interactions of human orthologues of the *Drosophila* proteins we developed the corresponding algorithm using PERL scripting language. The algorithm used the conversion table “*Drosophila* gene <-> human gene” produced as described above in this section (see Table S6) and the same online ID conversion tools. All records of physical interactions for the corresponding human proteins were extracted from BioGRID v.3.2.102 for *Homo sapiens* and the IDs were transformed to allow subsequent merging with the initial *Drosophila-*only network (see Fig. S1C) in the Cytoscape 2.8.3. To avoid resulting image overload (Fig. 3), multiple edges between the same proteins were merged into one. “Homodimerization” records obtained during “translation” process were excluded from the final result as they often were product of heterodimerization records between different human orthologues of the same *Drosophila* protein. The parameters of the random networks obtained during Monte Carlo simulation were calculated using GraphCrunch v.1.0 tool [68].

The programs created in-house will be available at the laboratory’s web-page (http://www.unil.ch/dpt/page85827.html).

## Supporting Information

Figure S1 **The complexity and connectivity of the** ***Drosophila*** **blisterome.** Blisterome constructed from the results of our RNAi screening only (A) increased upon addition of new components obtained from the published data (B) and further upon superimposition of physical interactions of their human orthologues (C). Each gene is an independent node, with edges between them being interactions of the genetic (cyan) or physical (red) nature, or being inferred from the physical interactions among their human orthologues (lilac). Nodes are color-coded, where genes disclosed in our RNAi-screening, extracted from published data, and their overlaps are shown as brown, yellow and orange nodes, respectively. Representative network parameters are placed below each corresponding graph. Note that the number of nodes in (A, 188) and (B, 327) is lower than the total number of genes in the gene lists used to construct these networks (208 and 358, respectively) as not all Drosophila genes have interaction reported in the BioGRID.
(PDF)

Figure S2 **Parameters characterizing the connectivity of a network are significantly higher (** ***p*** **<0.0001) for the** ***Drosophila*** **blisterome than for the set of 100 random networks (Monte Carlo simulations).** The “box & whiskers” graphs represent the median number of average neighbors per node (A), number of connected nodes (B) and clustering coefficient (C) for these networks, whiskers showing the 5–95 percentile and the black circles being outliers of this range. The values calculated for the *Drosophila* blisterome network are shown by dashed lines. Each simulated network was built for 358 randomly chosen *Drosophila* protein-coding genes. The statistical significance was evaluated by the one-sample Wilcoxon signed-rank test; the data were distributed non-normally according to the Kolmogorov-Smirnov test.
(PDF)

Table S1 **List of the** ***Drosophila*** **RNAi-lines used in the screening, 1709 RNAi-lines corresponding to 1573 genes in total, with the information on the transformant ID (TFID), construct ID (ConstrID), gene name, symbol, and synonyms, and described GO terms “biological process”, “molecular function”, and “cellular component”.** More information on the RNAi lines can be found at http://stockcenter.vdrc.at/control/main using the TFID; additional information on the targeted genes can be found at http://flybase.org/using the CG gene number. Information about relative GO terms can be found in appropriate columns.
(XLSX)

Table S2 **List of the RNAi lines producing wing blister phenotypes, 98 RNAi-lines corresponding to 91 genes in total, with the information on the transformant ID (TFID), construct ID (ConstrID), gene name, symbol, and synonyms, and described GO terms “biological process”, “molecular function”, and “cellular component”.** Different variations of the wing blister phenotype are indicated in column “Blister Phenotype”. Information whether genes from this list have previously been described as wing blister-causing can be found in the last column of the table (for citations refer to Table S5).
(XLSX)

Table S3 **List of the RNAi lines producing “burned wings” phenotypes, 123 RNAi-lines corresponding to 120 genes in total, with the information on the transformant ID (TFID), construct ID (ConstrID), gene name, symbol, and synonyms, and described GO terms “biological process”, “molecular function”, and “cellular component”.** Different variations of the “burned wings” phenotype are indicated in column “Burned wings Phenotype”. Information whether genes from this list have previously been described as wing blister-causing can be found in the last column of the table (for citations refer to Table S5).
(XLSX)

Table S4 **Over-represented “biological process”, “molecular function” and “cellular component” GO terms associated with genes from the merged list obtained from Tables S2 and S3.** GO enrichment analysis was performed using the DAVID bioinformatics web-tool with a set *p*-value cutoff ≤0.05. The complete list the screened genes (Table S1) was used as the background for analysis. Obtained *p*-values were corrected by the Benjamini correction technique provided by the DAVID program. The list of genes (their NCBI-Entrez numbers) associated with a particular GO term are provided in the last column.
(XLSX)

Table S5 **Genes extracted from published data described as wing blister-causing.** Information about genes’ CG number, name, symbol and function are indicated in the corresponding columns. For more information refer to the appropriate references listed below the table.
(DOCX)

Table S6 **Human orthologues found for the** ***Drosophila*** **blisterome components (Tables S2, S3, S5), with the information on the** ***Drosophila*** **gene CG number, gene name and symbol, as well as information on the resulting human orthologues.** Relation to diseases/disorders (see Fig. 5 in the main article) and human network of orthologues (see Fig. 4 in the main article) is indicated in the second last and last columns, respectively. For description and explanation of the orthology extraction algorithm see section Materials and Methods in the main article.
(XLSX)

Table S7 **Pathways implicated in governing of the** ***Drosophila*** **blisterome.** Among the total 358 proteins only 112 were assigned to different pathways according to a KEGG database, of which the Spliceosome, TGFβ and Proteasome pathways are among the most over-represented. The list of genes (their NCBI-Entrez numbers) associated with particular KEGG pathway term are placed in a last column of the table. Obtained *p*-values were corrected by the Benjamini correction technique provided by the DAVID tool and indicate that the three top terms are significantly enriched in the blisterome network as compared to the initial set of screened genes; the remaining terms are still shown to depict which main pathways the blisterome components belong to, even though they are not statistically over-represented.
(XLSX)

Table S8 **Human orthologues of the** ***Drosophila*** **blisterome components related to diseases and disorders.** 295 and 172 unique records were extracted from the OMIM catalog (columns 2–5) and KEGG database (columns 6–8) respectively, which were compiled then with overlapping records standing opposite each other. Classification of the diseases were taken from KEGG and ICD-10, maladies without available classification were classified manually.
(XLSX)

Table S9 **Distribution of human orthologues of the** ***Drosophila*** **blisterome components related to diseases and disorders among major systems of organs affected by specific maladies and associated with these genes.** One gene can be related to more than one disease, consequently affecting more than one system of organs (see Fig. 5 in the main article).
(XLSX)
