# Supplementary material for: Identification of Novel Elements of the Drosophila Blisterome Sheds Light on Potential Pathological Mechanisms of Several Human Diseases
Source: PLoS One. 2014 Jun 26;9(6):e101133. doi: 10.1371/journal.pone.0101133 (PMC4072764; doi:10.1371/journal.pone.0101133)
Supplement: Figure S1 — The complexity and connectivity of the Drosophila blisterome. Blisterome constructed from the results of our RNAi screening only (A) increased upon addition of new components obtained from the published data (B) and further upon superimposition of physical interactions of their human orthologues (C). Each gene is an independent node, with edges between them being interactions of the genetic (cyan) or physical (red) nature, or being inferred from the physical interactions among their human orthologues (lilac). Nodes are color-coded, where genes disclosed in our RNAi-screening, extracted from published data, and their overlaps are shown as brown, yellow and orange nodes, respectively. Representative network parameters are placed below each corresponding graph. Note that the number of nodes in (A, 188) and (B, 327) is lower than the total number of genes in the gene lists used to construct these networks (208 and 358, respectively) as not all Drosophila genes have interaction reported in the BioGRID. (PDF) [file pone.0101133.s001.pdf]

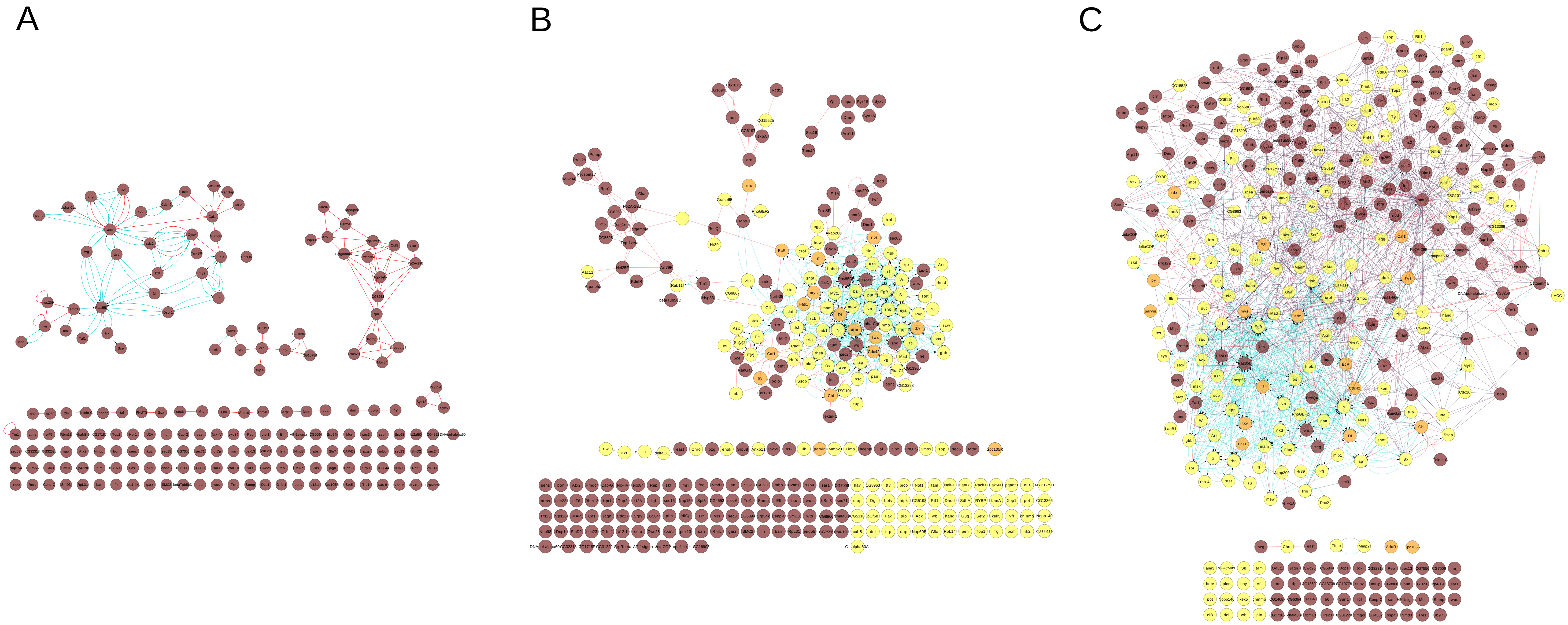

Clustering Coefficient: 0.12
Number of nodes: 188
Number of isolated nodes: 105
Av. number of neighbors: 1.16

Clustering Coefficient: 0.147
Number of nodes: 327
Number of isolated nodes: 143
Av. number of neighbors: 2.147

Clustering Coefficient: 0.228
Number of nodes: 358
Number of isolated nodes: 65
Av. number of neighbors: 6.37
