# Supplementary figures and images for: Identification of Novel Elements of the Drosophila Blisterome Sheds Light on Potential Pathological Mechanisms of Several Human Diseases
Source: PLoS One. 2014 Jun 26;9(6):e101133. doi: 10.1371/journal.pone.0101133 (PMC4072764; doi:10.1371/journal.pone.0101133)

**A**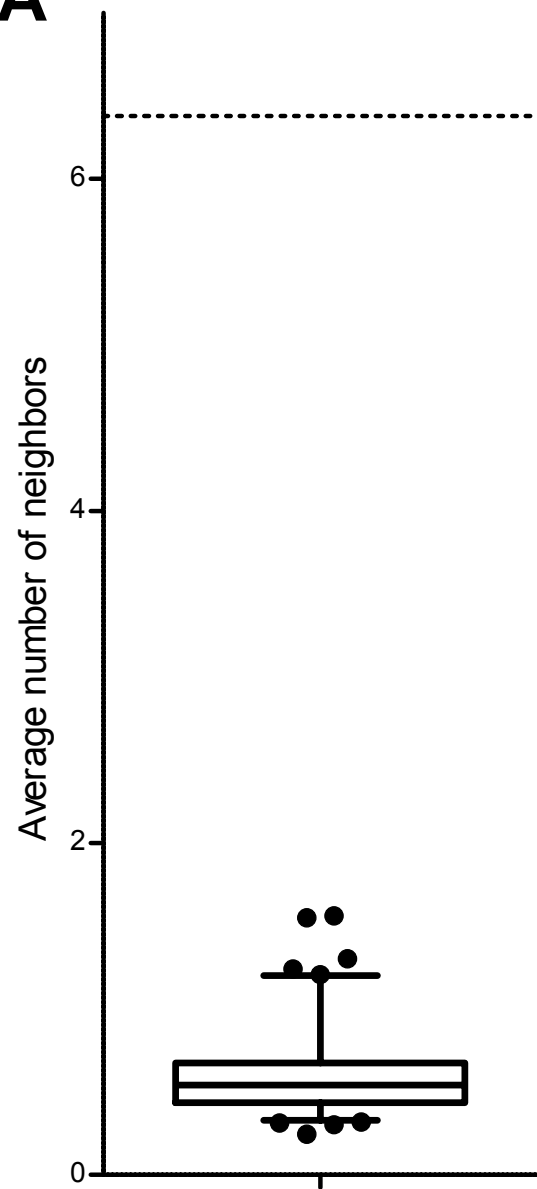**B**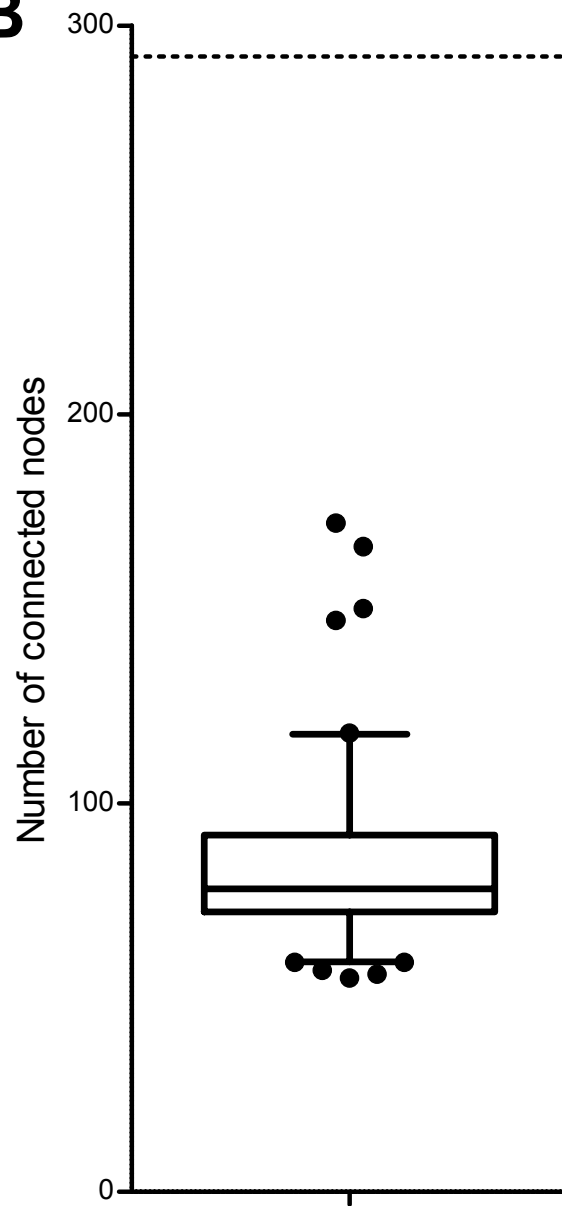**C**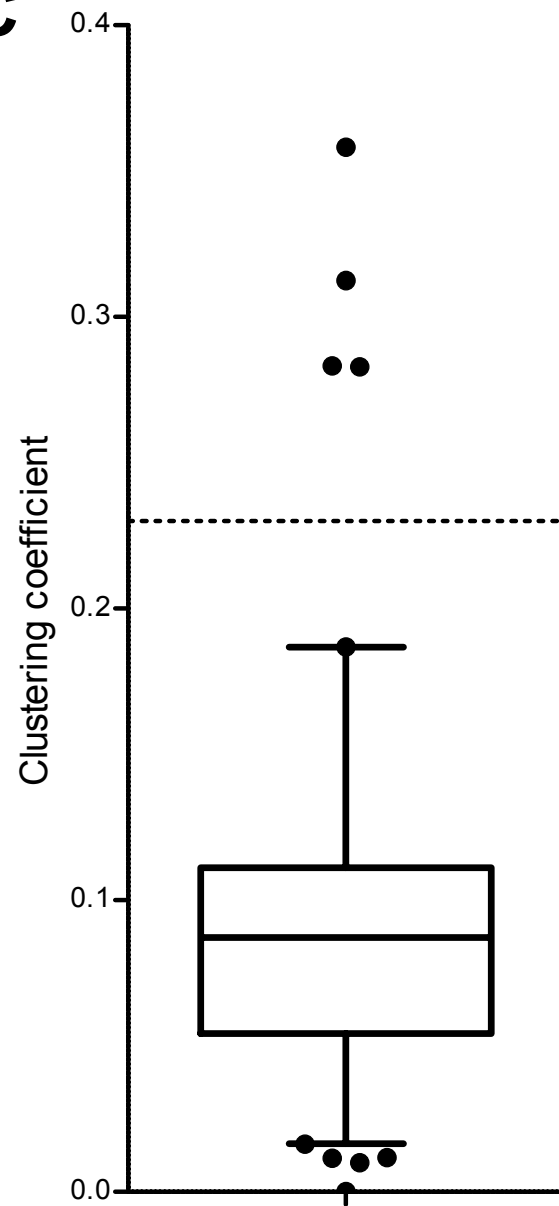

Supplement: Figure S2 — Parameters characterizing the connectivity of a network are significantly higher ( p <0.0001) for the Drosophila blisterome than for the set of 100 random networks (Monte Carlo simulations). The “box & whiskers” graphs represent the median number of average neighbors per node (A), number of connected nodes (B) and clustering coefficient (C) for these networks, whiskers showing the 5–95 percentile and the black circles being outliers of this range. The values calculated for the Drosophila blisterome network are shown by dashed lines. Each simulated network was built for 358 randomly chosen Drosophila protein-coding genes. The statistical significance was evaluated by the one-sample Wilcoxon signed-rank test; the data were distributed non-normally according to the Kolmogorov-Smirnov test. (PDF) [file pone.0101133.s002.pdf]
