# Supplementary material for: Identification of Novel Elements of the Drosophila Blisterome Sheds Light on Potential Pathological Mechanisms of Several Human Diseases
Source: PLoS One. 2014 Jun 26;9(6):e101133. doi: 10.1371/journal.pone.0101133 (PMC4072764; doi:10.1371/journal.pone.0101133)
Supplement: Table S5 — Genes extracted from published data described as wing blister-causing. Information about genes’ CG number, name, symbol and function are indicated in the corresponding columns. For more information refer to the appropriate references listed below the table. (DOCX) [file pone.0101133.s007.docx]

| Gene ID [ref.] | Name, symbol | Function |
| --- | --- | --- |
| CG1560 [[1](#_ENREF_1)] | myospheroid (mys, βPS) | adhesion |
| CG1771 [[1](#_ENREF_1)] | multiple edematous wings (mew, αPS1) | adhesion |
| CG9623 [[1](#_ENREF_1)] | inflated (if, αPS2) | adhesion |
| CG6831 [[2](#_ENREF_2)] | rhea, Talin | cytoskeletal anchor protein |
| CG9379 [[2](#_ENREF_2)] | blistery (by), Tensin | cytoskeletal crosslinker |
| CG7954 [[2](#_ENREF_2)] | steamer duck (stck), PINCH | cytoskeletal crosslinker |
| CG33196 [[2](#_ENREF_2)] | dumpy (dp) | extracellular matrix structural constituent |
| CG3619 [[2](#_ENREF_2)] | Delta (Dl) | ligand for Notch |
| CG8787 [[2](#_ENREF_2)] | Additional sex combs (Asx) | ubiquitin-specific protease activator activity; chromatin binding |
| CG1685 [[2](#_ENREF_2)] | penguin (pen) | RNA binding |
| CG8118 [[2](#_ENREF_2)] | mastermind (mam) | presumptive transcription factor |
| CG3411 [[2](#_ENREF_2)] | blistered (bs), SRF | transcription factor |
| CG18076 [[2](#_ENREF_2)] | short stop (shot) | cytoskeletal cross-linker protein |
| CG3541 [[2](#_ENREF_2)] | piopio (pio) | unknown |
| CG2467 [[3](#_ENREF_3)] | papillote (pot) | unknown |
| CG10293 [[4](#_ENREF_4)] | held out wings (how), struthio | mRNA binding |
| CG10504 [[5](#_ENREF_5)] | Integrin linked kinase (Ilk) | signaling |
| CG9031 [[6](#_ENREF_6)] | icarus (ics), RSU-1 | small GTPase regulator activity |
| CG9415 [[7](#_ENREF_7)] | X box binding protein-1 (Xbp1) | transcription factor |
| CG5841 [[7](#_ENREF_7)] | mind bomb 1 (mib1) | protein binding |
| CG5771 [[8](#_ENREF_8)] | Rab11 | signaling, vesicular trafficing |
| CG32528 [[9](#_ENREF_9)] | parvin | actin binding |
| CG4445 [[10](#_ENREF_10)] | polypeptide GalNAc transferase 3 (pgant3) | polypeptide N-acetylgalactosaminyltransferase activity; acetylgalactosaminyltransferase activity; transferase activity, transferring glycosyl groups |
| CG6741 [[11](#_ENREF_11)] | arc (a) | Scaffolding protein |
| CG4122 [[11](#_ENREF_11)] | silver (svr) | carboxypeptidase activity; metallocarboxypeptidase activity |
| CG8987 [[12](#_ENREF_12)] | tamas (tam) | DNA-directed DNA polymerase activity |
| CG8376 [[13](#_ENREF_13)] | apterous (ap) | transcription factor |
| CG3924 [[14](#_ENREF_14)] | Chip (Chi) | transcription factor |
| CG44425 [[15](#_ENREF_15)] | Beadex (Bx), dLMO | zinc ion binding |
| CG7187 [[16](#_ENREF_16)] | Sequence-specific single-stranded DNA-binding protein (Ssdp) | single-stranded DNA binding |
| CG6829 [[17](#_ENREF_17)] | Apaf-1-related-killer (Ark) | cysteine-type endopeptidase activator activity involved in apoptotic process |
| CG5123 [[18](#_ENREF_18)] | Wrinkled (W), Hid | programmed cell death |
| CG13366 [[19](#_ENREF_19)] |  | unknown |
| CG18214 [[20](#_ENREF_20)] | trio | signaling protein |
| CG12530 [[21](#_ENREF_21)] | Cdc42 | signaling |
| CG1716 [[22](#_ENREF_22)] | Set2 | chromatin enzyme |
| CG1765 [[23](#_ENREF_23)] | Ecdysone receptor (EcR) | Zinc finger transcription factor |
| CG14938 [[23](#_ENREF_23)] | crooked legs (crol) | transcription factor |
| CG2995 [[24](#_ENREF_24)] | G9a | histone-lysine N-methyltransferase activity |
| CG10236 [[25](#_ENREF_25)] | Laminin A (LanA), alpha3,5 chain | Extracellular matrix, Axon guidance |
| CG42677 [[26](#_ENREF_26)] | wing blister (wb), alpha1,2 chain | receptor binding |
| CG7123 [[27](#_ENREF_27)] | LanB1 | unknown |
| CG32417 [[28](#_ENREF_28)] | Myt1 | signaling |
| CG12190 [[29](#_ENREF_29)] | Ring and YY1 Binding Protein (RYBP) | zinc ion binding |
| CG5198 [[30](#_ENREF_30)] | hole-in-one (holn1) | unknown |
| CG18250 [[31](#_ENREF_31)] | Dystroglycan (Dg) | receptor |
| CG6281 [[32](#_ENREF_32)] | Tissue inhibitor of metalloproteases (Timp) | metalloendopeptidase inhibitor activity |
| CG12199 [[33](#_ENREF_33)] | kekkon5 (kek5) | unknown |
| CG8095 [[34](#_ENREF_34)] | scab (scb), alphaPS3 | cell surface adhesion protein |
| CG14026 [[35-37](#_ENREF_35)] | thickveins (tkv) | receptor kinase |
| CG2262 [[38](#_ENREF_38)] | Smad on X (Smox), dSmad2 | signal transduction |
| CG11940 [[39](#_ENREF_39)] | pico | protein phosphatase 1 binding |
| CG15792 [[40](#_ENREF_40)] | zipper (zip), Myo II | myosin light chain binding |
| CG9867 [[41](#_ENREF_41)] | EGF-domain O-GlcNAc transferase (Eogt) | protein O-GlcNAc transferase activity |
| CG7356 [[42](#_ENREF_42)] | Transglutaminase (Tg) | protein-glutamine gamma-glutamyltransferase activity |
| CG5994 [[43](#_ENREF_43)] | Negative elongation factor E (Nelf-E) | regulation of transcriptional elongation, RNA-binding protein |
| CG7421 [[44](#_ENREF_44)] | Nopp140 | unknown |
| CG6253 [[45](#_ENREF_45)] | Ribosomal protein L14 (RpL14) | structural constituent of ribosome |
| CG5441 [[46](#_ENREF_46)] | taxi (tx), dei | bHLH transcription factor binding; protein heterodimerization activity; E-box binding |
| CG13648 [[47](#_ENREF_47)] | tenectin (tnc) | integrin binding |
| CG3291 [[48](#_ENREF_48)] | pacman (pcm) | 5'-3' exoribonuclease activity |
| CG3903 [[49](#_ENREF_49)] | Gliotactin (Gli) | maintains blood-nerve barrier |
| CG4584 [[50](#_ENREF_50)] | Deoxyuridine triphosphatase (dUTPase) | dUTP diphosphatase activity |
| CG8171 [[51](#_ENREF_51)] | double parked (dup) | required for DNA replication |
| CG17246 [[51](#_ENREF_51)] | Succinate dehydrogenase A (SdhA) | succinate dehydrogenase (ubiquinone) activity (general metabolism) |
| CG15525 [[51](#_ENREF_51)] |  | unknown (protein with conserved structural motives) |
| CG8963 [[51](#_ENREF_51)] |  | RNA binding; DNA binding |
| CG9968 [[51](#_ENREF_51)] | Annexin B11 (AnxB11) | actin binding |
| CG9310 [[51](#_ENREF_51)] | Hepatocyte nuclear factor 4 (Hnf4) | Transcription factor |
| CG12537 [[51](#_ENREF_51)] | roadkill (rdx) | protein binding |
| CG13388 [[51](#_ENREF_51)] | A kinase anchor protein 200 (Akap200) | protein kinase A binding (cell signaling) |
| CG17090 [[51](#_ENREF_51)] | homeodomain interacting protein kinase (hipk) | signaling |
| CG6588 [[51](#_ENREF_51)] | Fasciclin 1 (Fas1) | cell adhesion molecule binding |
| CG3167 [[51](#_ENREF_51)] | MAN1 | unknown (protein with conserved structural motives) |
| CG11290 [[51](#_ENREF_51)] | enoki mushroom (enok) | histone acetyltransferase activity (transcription factor) |
| CG9712 [[51](#_ENREF_51)] | tumor suppressor protein 101 (TSG101) | vesicular transport protein, signaling |
| CG3905 [[51](#_ENREF_51)] | Suppressor of zeste 2 (Su(z)2) | DNA binding |
| CG6582 [[52](#_ENREF_52)] | Aac11 | negative regulation of apoptotic process; neurogenesis |
| CG33950 [[53](#_ENREF_53)] | terribly reduced optic lobes (trol) | unknown |
| CG18361 [[53](#_ENREF_53)] | dishevelled (dsh) | signal transduction |
| CG18572 [[53](#_ENREF_53)] | rudimentary (r) | dihydroorotase activity; aspartate carbamoyltransferase activity; carbamoyl-phosphate synthase (glutamine-hydrolyzing) activity |
| CG6146 [[53](#_ENREF_53)] | Topoisomerase 1 (Top 1) | DNA topoisomerase activity; DNA topoisomerase type I activity; protein kinase activity |
| CG6998 [[53](#_ENREF_53)] | cut up (ctp) | dynein intermediate chain binding; protein binding; protein homodimerization activity |
| CG4220 [[54](#_ENREF_54)] | elbow B (elB) | putative transcription factor |
| CG32443 [[55](#_ENREF_55)] | Polycomb (Pc) | transcription factor |
| CG8491 [[55](#_ENREF_55)] | kohtalo (kto) | protein binding; RNA polymerase II transcription cofactor activity |
| CG9936 [[55](#_ENREF_55)] | skuld (skd) | protein binding; RNA polymerase II transcription cofactor activity |
| CG6502 [[55](#_ENREF_55)] | Enhancer of zeste (E(z)) | transcription factor, enzyme |
| CG3936 [[56](#_ENREF_56)] | Notch (N) | receptor, lateral inhibition |
| CG3830 [[57](#_ENREF_57)] | vestigial (vg) | Presumptive transcription factor |
| CG11312 [[58](#_ENREF_58)] | inscuteable (insc) | Links cytoskeleton to spindle-orientation and protein subcellular distribution |
| CG10117 [[59](#_ENREF_59)] | tout-velu (ttv) | enzyme |
| CG15110 [[59](#_ENREF_59)] | brother of tout-velu (botv) | acetylglucosaminyltransferase activity |
| CG8433 [[59](#_ENREF_59)] | Ext2 | protein binding |
| CG11198 [[60](#_ENREF_60)] | Acetyl-CoA carboxylase (ACC) | acetyl-CoA carboxylase activity |
| CG10712 [[60](#_ENREF_60)] | Chromator (Chro) | histone binding |
| CG10955 [[60](#_ENREF_60)] | Rtf1 | chromatin modification, signaling |
| CG4236 [[60](#_ENREF_60)] | Chromatin assembly factor 1 subunit (Caf1) | protein binding; nucleosome binding; histone acetyltransferase binding; histone binding; protein homodimerization activity; histone |
| CG14813 [[60](#_ENREF_60)] | δ-coatomer protein (δCOP) | unknown |
| CG9476 [[60](#_ENREF_60)] | α-Tubulin at 85E (αTub85E) | structural constituent of cytoskeleton |
| CG31666 [[60](#_ENREF_60)] | Chronologically inappropriate morphogenesis (chinmo) | transcription factor |
| CG13298 [[60](#_ENREF_60)] |  | mRNA binding |
| CG11451 [[60](#_ENREF_60)] | Spc105-related (Spc105R) | unknown |
| CG34407 [[60](#_ENREF_60)] | Not1 | protein binding |
| CG7809 [[61](#_ENREF_61)] | Grasp65 | signaling |
| CG33197 [[62](#_ENREF_62)] | mbl | DNA binding; nucleic acid binding |
| CG3333 [[63](#_ENREF_63)] | Nucleolar protein at 60B (Nop60B) | enzyme |
| CG7904 [[64](#_ENREF_64)] | punt (put) | receptor - serine/threonine kinase |
| CG9741 [[65](#_ENREF_65)] | Dihydroorotate dehydrogenase (Dhod) | dihydroorotate dehydrogenase activity |
| CG10619 [[66](#_ENREF_66)] | tailup (tup), isl | Transcription factor |
| CG7111 [[67](#_ENREF_67)] | Receptor of activated protein kinase C 1 (Rack1) | protein kinase C binding |
| CG9635 [[68](#_ENREF_68)] | Rho guanine nucleotide exchange factor 2 (RhoGEF2) | signaling |
| CG12196 [[69](#_ENREF_69)] | eggless (egg), dEset | chromatin component |
| CG14992 [[70](#_ENREF_70)] | Activated Cdc42 kinase (Ack) | SH2 domain binding; protein tyrosine kinase activity |
| CG5110 [[71](#_ENREF_71)] | MP1 | SH3/SH2 adaptor activity |
| CG4316 [[53](#_ENREF_53)] | Stubble | serine-type endopeptidase activity |
| CG43122 [[72](#_ENREF_72)] | capicua (cic) | sequence-specific DNA binding; repressing transcription factor binding |
| CG4370 [[73](#_ENREF_73)] | Inwardly rectifying potassium channel 2 (Irk2) | inward rectifier potassium channel activity |
| CG10079 [[74](#_ENREF_74)] | Epidermal growth factor receptor (Egfr) | transmembrane signaling |
| CG3352 [[75](#_ENREF_75)] | fat (ft) | transmembrane receptor |
| CG2096 [[76](#_ENREF_76)] | flapwing (flw) | signaling |
| CG6964 [[77](#_ENREF_77)] | Grunge (Gug), Atro | Transcription co-factor |
| CG34403 [[78](#_ENREF_78)] | pangolin (pan) | transcription factor |
| CG9753 [[79](#_ENREF_79)] | Adenosine receptor (AdoR) | G-protein coupled adenosine receptor activity |
| CG8676 [[51](#_ENREF_51), [80](#_ENREF_80)] | Hormone receptor-like in 39 (Hr39) | Transcription factor |
| CG2835 [[81](#_ENREF_81)] | G protein α s subunit (Gαs)* | GTPase activity |
| CG8556 [[80](#_ENREF_80)] | Rac2 | GTPase activity |
| CG1004 [[82](#_ENREF_82)] | rhomboid (rho) | intramembrane serine protease |
| CG33166 [[83](#_ENREF_83)] | stem cell tumor (stet), rho2 | enzyme |
| CG1214 [[84](#_ENREF_84)] | roughoid (ru), rho3 | unknown |
| CG1697 [[83](#_ENREF_83)] | rhomboid-4 (rho-4) | serine-type peptidase activity |
| CG32179 [[83](#_ENREF_83)] | Keren (Krn) | ligand |
| CG6863 [[85](#_ENREF_85)] | tolkin (tok) | protease |
| CG10023 [[86](#_ENREF_86)] | Focal adhesion kinase (Fak) | signal transduction |
| CG4319 [[52](#_ENREF_52)] | reaper (rpr) | programmed cell death |
| CG6376 [[52](#_ENREF_52)] | E2F transcription factor (E2f) | transcription factor |
| CG12399 [[87](#_ENREF_87)] | Mothers against dpp (Mad) | TGF beta signal transduction |
| CG7935 [[88](#_ENREF_88)] | moleskin (msk) | signaling |
| CG31794 [[89](#_ENREF_89)] | Paxillin (Pax) | zinc ion binding |
| CG1794 [[90](#_ENREF_90)] | Matrix metalloproteinase 2 (Mmp2) | metalloendopeptidase activity |
| CG1098 [[91](#_ENREF_91)] | MLF1-adaptor molecule (Madm) | protein serine/threonine kinase activity |
| CG12701 [[92](#_ENREF_92)] | vielfaltig (vfl) | transcription factor |
| CG7926 [[93](#_ENREF_93)] | Axin (Axn) | scaffolding protein |
| CG11579 [[93](#_ENREF_93)] | armadillo (arm) | cytoskeletonal element |
| CG7892 [[93](#_ENREF_93)] | nemo (nmo) | signaling |
| CG8224 [[38](#_ENREF_38)] | baboon (babo)* | receptor tyrosine kinase |
| CG9885 [[36](#_ENREF_36)] | decapentaplegic (dpp) | secreted morphogen |
| CG5562 [[36](#_ENREF_36)] | glass bottom boat (gbb) | ligand |
| CG1891 [[36](#_ENREF_36)] | saxophone (sax)* | surface receptor |
| CG9311 [[94](#_ENREF_94)] | myopic (mop) | protein tyrosine phosphatase activity |
| CG9126 [[95](#_ENREF_95)] | Stromal interaction molecule (Stim) | calcium-induced calcium release activity; store-operated calcium channel activity |
| CG12559 [[96](#_ENREF_96)] | rolled (rl)* | serine/threonine protein kinase |
| CG10275 [[97](#_ENREF_97)] | kon-tiki (kon) | receptor |
| CG4379 [[98](#_ENREF_98)] | cAMP-dependent protein kinase 1 (Pka-C1) | Signal transduction |
| CG6235 [[99](#_ENREF_99)] | twins (tws) | serine/threonine protein phosphatase |
| CG4385 [[100](#_ENREF_100)] | Star (S) | modulation of EGF-R signaling |
| CG10491 [[101](#_ENREF_101)] | vein (vn)* | ligand for EGF receptor |
| CG5920 [[102](#_ENREF_102)] | Ribosomal protein S2 (RpS2) | structural constituent of ribosome |
| CG32575 [[102](#_ENREF_102)] | hangover (hang) | nucleic acid binding |
| CG8222 [[103](#_ENREF_103)] | PDGF- and VEGF-receptor related (Pvr) | receptor tyrosine kinase |
| CG8019 [[104](#_ENREF_104)] | haywire (hay) | helicase activity; ATP-dependent 3'-5' DNA helicase activity |
| CG9554 [[105](#_ENREF_105)] | eyes absent (eya) | transcription factor and enzyme |
| CG12085 [[106](#_ENREF_106)] | poly U binding factor 68kD (pUf68), hfp | mRNA splicing factor |
| CG31695 [[37](#_ENREF_37)] | screw (scw) | DPP signaling |
| CG6896 [[107](#_ENREF_107)] | MYPT-75D* | protein phosphatase 1 binding; myosin phosphatase regulator activity |
| CG11614 [[108](#_ENREF_108)] | naked cuticle (nkd) | signaling |
| CG1401 [[109](#_ENREF_109)] | Cullin-5 (Cul-5) | ubiquitin protein ligase binding |

1. *Brabant M. C., Fristrom D., Bunch T. A., Baker S. E., and Brower D. L.* The PS Integrins Are Required for a Regulatory Event during Drosophila Wing Morphogenesisa // Annals of the New York Academy of Sciences. — 1998.— **857**, N 1.— P. 99-109.

2. *Prout M., Damania Z., Soong J., Fristrom D., and Fristrom J. W.* Autosomal Mutations Affecting Adhesion Between Wing Surfaces in Drosophila melanogaster // Genetics. — 1997.— **146**, N 1.— P. 275-285.

3. *Walsh E. P. and Brown N. H.* A Screen to Identify Drosophila Genes Required for Integrin-Mediated Adhesion // Genetics. — 1998.— **150**, N 2.— P. 791-805.

4. *Lo P. C. H. and Frasch M.* A Novel KH-Domain Protein Mediates Cell Adhesion Processes inDrosophila // Developmental Biology. — 1997.— **190**, N 2.— P. 241-256.

5. *Zervas C. G., Gregory S. L., and Brown N. H.* Drosophila Integrin-Linked Kinase Is Required at Sites of Integrin Adhesion to Link the Cytoskeleton to the Plasma Membrane // J Cell Biol. — 2001.— **152**, N 5.— P. 1007-1018.

6. *Kadrmas J. L., Smith M. A., Clark K. A., Pronovost S. M., Muster N., Yates J. R., and Beckerle M. C.* The integrin effector PINCH regulates JNK activity and epithelial migration in concert with Ras suppressor 1 // J Cell Biol. — 2004.— **167**, N 6.— P. 1019-1024.

7. *Mummery-Widmer J. L., Yamazaki M., Stoeger T., Novatchkova M., Bhalerao S., Chen D., Dietzl G., Dickson B. J., and Knoblich J. A.* Genome-wide analysis of Notch signalling in Drosophila by transgenic RNAi // Nature. — 2009.— **458**, N 7241.— P. 987-992.

8. *Bhuin T. and Roy J. K.* Rab11 is required for cell adhesion, maintenance of cell shape and actin-cytoskeleton organization during Drosophila wing development // Int J Dev Biol. — 2011.— **55**, N 3.— P. 269-279.

9. *Vakaloglou K. M., Chountala M., and Zervas C. G.* Functional analysis of parvin and different modes of IPP-complex assembly at integrin sites during Drosophila development // Journal of Cell Science. — 2012.— **125**, N 13.— P. 3221-3232.

10. *Zhang L., Zhang Y., and Hagen K. G. T.* A Mucin-type O-Glycosyltransferase Modulates Cell Adhesion during Drosophila Development // J. Biol. Chem. — 2008.— **283**, N 49.— P. 34076-34086.

11. *Goldschmidt R.* A Mutant of Drosophila Melanogaster Resembling the So-Called Unstable Genes of Drosophila Virilis // P Natl Acad Sci USA. — 1943.— **29**, N 7.— P. 203-206.

12. *Ashburner M., Misra S., Roote J., Lewis S. E., Blazej R., Davis T., Doyle C., Galle R., George R., Harris N., Hartzell G., Harvey D., Hong L., Houston K., Hoskins R., Johnson G., Martin C., Moshrefi A., Palazzolo M., Reese M. G., Spradling A., Tsang G., Wan K., Whitelaw K., Kimmel B., Celniker S., and Rubin G. M.* An Exploration of the Sequence of a 2.9-Mb Region of the Genome of Drosophila melanogaster: The Adh Region // Genetics. — 1999.— **153**, N 1.— P. 179-219.

13. *Stevens M. E. and Bryant P. J.* Apparent Genetic Complexity Generated by Developmental Thresholds: The Apterous Locus in Drosophila Melanogaster // Genetics. — 1985.— **110**, N 2.— P. 281-297.

14. *Milan M. and Cohen S. M.* Temporal regulation of apterous activity during development of the Drosophila wing // Development. — 2000.— **127**, N 14.— P. 3069-3078.

15. *Jack J. and DeLotto Y.* Effect of wing scalloping mutations on cut expression and sense organ differentiation in the Drosophila wing margin // Genetics. — 1992.— **131**, N 2.— P. 353-363.

16. *Meyel D. J. v., Thomas J. B., and Agulnick A. D.* Ssdp proteins bind to LIM-interacting co-factors and regulate the activity of LIM-homeodomain protein complexes in vivo // Development. — 2003.— **130**, N 9.— P. 1915-1925.

17. *Rodriguez A., Oliver H., Zou H., Chen P., Wang X., and Abrams J. M.* Dark is a Drosophila homologue of Apaf-1/CED-4 and functions in an evolutionarily conserved death pathway // Nature Cell Biology. — 1999.— **1**, N 5.— P. 272-279.

18. *White K.* Cell death: Drosophila Apaf-1 — no longer in the (d)Ark // Current Biology. — 2000.— **10**, N 4.— P. R167-R169.

19. *Saadi I., Alkuraya Fowzan S., Gisselbrecht Stephen S., Goessling W., Cavallesco R., Turbe-Doan A., Petrin Aline L., Harris J., Siddiqui U., Grix Jr Arthur W., Hove Hanne D., Leboulch P., Glover Thomas W., Morton Cynthia C., Richieri-Costa A., Murray Jeffrey C., Erickson Robert P., and Maas Richard L.* Deficiency of the Cytoskeletal Protein SPECC1L Leads to Oblique Facial Clefting // The American Journal of Human Genetics. — 2011.— **89**, N 1.— P. 44-55.

20. *Liebl E. C., Forsthoefel D. J., Franco L. S., Sample S. H., Hess J. E., Cowger J. A., Chandler M. P., Shupert A. M., and Seeger M. A.* Dosage-Sensitive, Reciprocal Genetic Interactions between the Abl Tyrosine Kinase and the Putative GEF trio Reveal trio's Role in Axon Pathfinding // Neuron. — 2000.— **26**, N 1.— P. 107-118.

21. *Eaton S., Auvinen P., Luo L., Jan Y. N., and Simons K.* CDC42 and Rac1 control different actin-dependent processes in the Drosophila wing disc epithelium // J Cell Biol. — 1995.— **131**, N 1.— P. 151-164.

22. *Stabell M., Larsson J., Aalen R. B., and Lambertsson A.* Drosophila dSet2 functions in H3-K36 methylation and is required for development // Biochemical and Biophysical Research Communications. — 2007.— **359**, N 3.— P. 784-789.

23. *D'Avino P. P. and Thummel C. S.* The Ecdysone Regulatory Pathway Controls Wing Morphogenesis and Integrin Expression during Drosophila Metamorphosis // Developmental Biology. — 2000.— **220**, N 2.— P. 211-224.

24. *Stabell M., Eskeland R., Bj?rkmo M., Larsson J., Aalen R. B., Imhof A., and Lambertsson A.* The Drosophila G9a gene encodes a multi-catalytic histone methyltransferase required for normal development // Nucleic Acids Research. — 2006.— **34**, N 16.— P. 4609-4621.

25. *Henchcliffe C., Garcia-Alonso L., Tang J., and Goodman C. S.* Genetic analysis of laminin A reveals diverse functions during morphogenesis in Drosophila // Development. — 1993.— **118**, N 2.— P. 325-337.

26. *Martin D., Zusman S., Li X., Williams E. L., Khare N., DaRocha S., Chiquet-Ehrismann R., and Baumgartner S.* wing blister, A New Drosophila Laminin α Chain Required for Cell Adhesion and Migration during Embryonic and Imaginal Development // J Cell Biol. — 1999.— **145**, N 1.— P. 191-201.

27. *Urbano J. M., Torgler C. N., Molnar C., Tepass U., López-Varea A., Brown N. H., Celis J. F. d., and Martín-Bermudo M. D.* Drosophila laminins act as key regulators of basement membrane assembly and morphogenesis // Development. — 2009.— **136**, N 24.— P. 4165-4176.

28. *Jin Z., Homola E., Tiong S., and Campbell S. D.* Drosophila Myt1 Is the Major Cdk1 Inhibitory Kinase for Wing Imaginal Disc Development // Genetics. — 2008.— **180**, N 4.— P. 2123-2133.

29. *González I., Aparicio R., and Busturia A.* Functional Characterization of the dRYBP Gene in Drosophila // Genetics. — 2008.— **179**, N 3.— P. 1373-1388.

30. *Geiger J. A., Carvalho L., Campos I., Santos A. C., and Jacinto A.* Hole-in-One Mutant Phenotypes Link EGFR/ERK Signaling to Epithelial Tissue Repair in Drosophila // PloS one. — 2011.— **6**, N 11.—

31. *Ueyama M., Akimoto Y., Ichimiya T., Ueda R., Kawakami H., Aigaki T., and Nishihara S.* Increased Apoptosis of Myoblasts in Drosophila Model for the Walker-Warburg Syndrome // PloS one. — 2010.— **5**, N 7.—

32. *Godenschwege T. A., Pohar N., Buchner S., and Buchner E.* Inflated wings, tissue autolysis and early death in tissue inhibitor of metalloproteinases mutants of Drosophila // European Journal of Cell Biology. — 2000.— **79**, N 7.— P. 495-501.

33. *Evans T. A., Haridas H., and Duffy J. B.* Kekkon5 is an extracellular regulator of BMP signaling // Developmental Biology. — 2009.— **326**, N 1.— P. 36-46.

34. *Araujo H., Negreiros E., and Bier E.* Integrins modulate Sog activity in the Drosophila wing // Development. — 2003.— **130**, N 16.— P. 3851-3864.

35. *Terracol R. and Lengyel J. A.* The thick veins gene of Drosophila is required for dorsoventral polarity of the embryo // Genetics. — 1994.— **138**, N 1.— P. 165-178.

36. *Haerry T. E., Khalsa O., O'Connor M. B., and Wharton K. A.* Synergistic signaling by two BMP ligands through the SAX and TKV receptors controls wing growth and patterning in Drosophila // Development. — 1998.— **125**, N 20.— P. 3977-3987.

37. *Nguyen M., Park S., Marqués G., and Arora K.* Interpretation of a BMP Activity Gradient in Drosophila Embryos Depends on Synergistic Signaling by Two Type I Receptors, SAX and TKV // Cell. — 1998.— **95**, N 4.— P. 495-506.

38. *Peterson A. J., Jensen P. A., Shimell M., Stefancsik R., Wijayatonge R., Herder R., Raftery L. A., and O'Connor M. B.* R-Smad Competition Controls Activin Receptor Output in Drosophila // PloS one. — 2012.— **7**, N 5.—

39. *Thompson B. J.* Mal/SRF Is Dispensable for Cell Proliferation in Drosophila // PloS one. — 2010.— **5**, N 4.—

40. *Franke J. D., Montague R. A., and Kiehart D. P.* Nonmuscle myosin II is required for cell proliferation, cell sheet adhesion and wing hair morphology during wing morphogenesis // Developmental Biology. — 2010.— **345**, N 2.— P. 117-132.

41. *Sakaidani Y., Ichiyanagi N., Saito C., Nomura T., Ito M., Nishio Y., Nadano D., Matsuda T., Furukawa K., and Okajima T.* O-linked-N-acetylglucosamine modification of mammalian Notch receptors by an atypical O-GlcNAc transferase Eogt1 // Biochemical and Biophysical Research Communications. — 2012.— **419**, N 1.— P. 14-19.

42. *Shibata T., Ariki S., Shinzawa N., Miyaji R., Suyama H., Sako M., Inomata N., Koshiba T., Kanuka H., and Kawabata S.-i.* Protein Crosslinking by Transglutaminase Controls Cuticle Morphogenesis in Drosophila // PloS one. — 2010.— **5**, N 10.—

43. *Enerly E., Larsson J., and Lambertsson A.* Reverse genetics in drosophila: From sequence to phenotype using UAS-RNAi transgenic flies // genesis. — 2002.— **34**, N 1-2.— P. 152-155.

44. *Cui Z. and DiMario P. J.* RNAi Knockdown of Nopp140 Induces Minute-like Phenotypes in Drosophila // Mol. Biol. Cell. — 2007.— **18**, N 6.— P. 2179-2191.

45. *Enerly E., Larsson J., and Lambertsson A.* Silencing the Drosophila ribosomal protein L14 gene using targeted RNA interference causes distinct somatic anomalies // Gene. — 2003.— **320**, N P. 41-48.

46. *Egoz-Matia N., Nachman A., Halachmi N., Toder M., Klein Y., and Salzberg A.* Spatial regulation of cell adhesion in the Drosophila wing is mediated by Delilah, a potent activator of βPS integrin expression // Developmental Biology. — 2011.— **351**, N 1.— P. 99-109.

47. *Fraichard S., Bougé A.-L., Kendall T., Chauvel I., Bouhin H., and Bunch T. A.* Tenectin is a novel αPS2βPS integrin ligand required for wing morphogenesis and male genital looping in Drosophila // Developmental Biology. — 2010.— **340**, N 2.— P. 504-517.

48. *Grima D. P., Sullivan M., Zabolotskaya M. V., Browne C., Seago J., Wan K. C., Okada Y., and Newbury S. F.* The 5′–3′ exoribonuclease pacman is required for epithelial sheet sealing in Drosophila and genetically interacts with the phosphatase puckered // Biology of the Cell. — 2008.— **100**, N 12.— P. 687-701.

49. *Venema D. R., Zeev-Ben-Mordehai T., and Auld V. J.* Transient apical polarization of Gliotactin and Coracle is required for parallel alignment of wing hairs in Drosophila // Developmental Biology. — 2004.— **275**, N 2.— P. 301-314.

50. *Muha V., Horvath A., Bekesi A., Pukancsik M., Hodoscsek B., Merenyi G., Rona G., Batki J., Kiss I., Jankovics F., Vilmos P., Erdelyi M., and Vertessy B. G.* Uracil-Containing DNA in Drosophila: Stability, Stage-Specific Accumulation, and Developmental Involvement // PLoS Genet. — 2012.— **8**, N 6.—

51. *Molnar C., Casado M., López-Varea A., Cruz C., and Celis J. F. d.* Genetic Annotation of Gain-Of-Function Screens Using RNA Interference and in Situ Hybridization of Candidate Genes in the Drosophila Wing // Genetics. — 2012.— **192**, N 2.— P. 741-752.

52. *Morris E. J., Michaud W. A., Ji J.-Y., Moon N.-S., Rocco J. W., and Dyson N. J.* Functional Identification of Api5 as a Suppressor of E2F-Dependent Apoptosis In Vivo // PLoS Genet. — 2006.— **2**, N 11.—

53. *McQuilton P., St. Pierre S. E., Thurmond J., and Consortium t. F.* FlyBase 101 – the basics of navigating FlyBase // Nucleic Acids Research. — 2012.— **40**, N D1.— P. D706-D714.

54. Ashburner M., *[Entry for el.]*, 1992. p. 194-195.

55. *Alonso A. G. d. A., Gutiérrez L., Fritsch C., Papp B., Beuchle D., and Müller J.* A Genetic Screen Identifies Novel Polycomb Group Genes in Drosophila // Genetics. — 2007.— **176**, N 4.— P. 2099-2108.

56. *Bateman A. J.* [New mutants report.] // Drosophila Information Service. — 1950.— **24**, N P. 54-56.

57. *Alexandrov I. D. and Alexandrova M. V.* Report of new mutants // Drosophila Information Service. — 1987.— **66**, N P. 185-187.

58. *Kraut R. and Campos-Ortega J. A.* inscuteable,A Neural Precursor Gene ofDrosophila,Encodes a Candidate for a Cytoskeleton Adaptor Protein // Developmental Biology. — 1996.— **174**, N 1.— P. 65-81.

59. *Han C., Belenkaya T. Y., Khodoun M., Tauchi M., Lin X., and Lin X.* Distinct and collaborative roles of Drosophila EXT family proteins in morphogen signalling and gradient formation // Development. — 2004.— **131**, N 7.— P. 1563-1575.

60. *Friedman A. A., Tucker G., Singh R., Yan D., Vinayagam A., Hu Y., Binari R., Hong P., Sun X., Porto M., Pacifico S., Murali T., Finley R. L., Asara J. M., Berger B., and Perrimon N.* Proteomic and Functional Genomic Landscape of Receptor Tyrosine Kinase and Ras to Extracellular Signal-Regulated Kinase Signaling // Sci. Signal. — 2011.— **4**, N 196.—

61. *Schotman H., Karhinen L., and Rabouille C.* dGRASP-Mediated Noncanonical Integrin Secretion Is Required for Drosophila Epithelial Remodeling // Developmental Cell. — 2008.— **14**, N 2.— P. 171-182.

62. *Prokopenko S. N., He Y., Lu Y., and Bellen H. J.* Mutations Affecting the Development of the Peripheral Nervous System in Drosophila: A Molecular Screen for Novel Proteins // Genetics. — 2000.— **156**, N 4.— P. 1691-1715.

63. *Tortoriello G., de Celis J. F., and Furia M.* Linking pseudouridine synthases to growth, development and cell competition // FEBS Journal. — 2010.— **277**, N 15.— P. 3249-3263.

64. *Penton A. and Hoffmann F. M.* Decapentaplegic restricts the domain of wingless during Drosophila limb patterning // Nature. — 1996.— **382**, N 6587.— P. 162-165.

65. *Löffler M., Knecht W., Rawls J., Ullrich A., and Dietz C.* Drosophila melanogaster dihydroorotate dehydrogenase: the N-terminus is important for biological function in vivo but not for catalytic properties in vitro // Insect Biochemistry and Molecular Biology. — 2002.— **32**, N 9.— P. 1159-1169.

66. *O'Keefe D. D., Thor S., and Thomas J. B.* Function and specificity of LIM domains in Drosophila nervous system and wing development // Development. — 1998.— **125**, N 19.— P. 3915-3923.

67. *Kadrmas J. L., Smith M. A., Pronovost S. M., and Beckerle M. C.* Characterization of RACK1 function in Drosophila development // Developmental Dynamics. — 2007.— **236**, N 8.— P. 2207-2215.

68. *Nikolaidou K. K. and Barrett K.* A Rho GTPase Signaling Pathway Is Used Reiteratively in Epithelial Folding and Potentially Selects the Outcome of Rho Activation // Current Biology. — 2004.— **14**, N 20.— P. 1822-1826.

69. *Stabell M., Bjørkmo M., Aalen R. B., and Lambertsson A.* The Drosophila SET domain encoding gene dEset is essential for proper development // Hereditas. — 2006.— **143**, N 2006.— P. 177-188.

70. *Sem K. P., Zahedi B., Tan I., Deak M., Lim L., and Harden N.* ACK Family Tyrosine Kinase Activity Is a Component of Dcdc42 Signaling during Dorsal Closure in Drosophila melanogaster // Molecular and Cellular Biology. — 2002.— **22**, N 11.— P. 3685-3697.

71. *Mouchel-Vielh E., Bloyer S., Salvaing J., Randsholt N. B., and Peronnet F.* Involvement of the MP1 scaffold protein in ERK signaling regulation during Drosophila wing development // Genes to Cells. — 2008.— **13**, N 11.— P. 1099-1111.

72. *Goff D. J., Nilson L. A., and Morisato D.* Establishment of dorsal-ventral polarity of the Drosophila egg requires capicua action in ovarian follicle cells // Development. — 2001.— **128**, N 22.— P. 4553-4562.

73. *Dahal G. R., Rawson J., Gassaway B., Kwok B., Tong Y., Ptacek L. J., and Bates E.* An inwardly rectifying K+ channel is required for patterning // Development (Cambridge, England). — 2012.— **139**, N 19.— P. 3653-3664.

74. *Diaz-Benjumea F. J. and Garcia-Bellido A.* Behaviour of Cells Mutant for an EGF Receptor Homologue of Drosophila in Genetic Mosaics // Proc. R. Soc. Lond. B. — 1990.— **242**, N 1303.— P. 36-44.

75. *Garoia F., Guerra D., Pezzoli M. C., López-Varea A., Cavicchi S., Garcı, and ́a-Bellido A.* Cell behaviour of Drosophila fat cadherin mutations in wing development // Mechanisms of Development. — 2000.— **94**, N 1–2.— P. 95-109.

76. *Raghavan S., Williams I., Aslam H., Thomas D., Szöőr B., Morgan G., Gross S., Turner J., Fernandes J., VijayRaghavan K., and Alphey L.* Protein phosphatase 1β is required for the maintenance of muscle attachments // Current Biology. — 2000.— **10**, N 5.— P. 269-272.

77. *Fanto M., Clayton L., Meredith J., Hardiman K., Charroux B., Kerridge S., and McNeill H.* The tumor-suppressor and cell adhesion molecule Fat controls planar polarity via physical interactions with Atrophin, a transcriptional co-repressor // Development. — 2003.— **130**, N 4.— P. 763-774.

78. *Kiger J. A., Natzle J. E., and Green M. M.* Hemocytes are essential for wing maturation in Drosophila melanogaster // P Natl Acad Sci USA. — 2001.— **98**, N 18.— P. 10190-10195.

79. *Dolezelova E., Nothacker H.-P., Civelli O., Bryant P. J., and Zurovec M.* A Drosophila adenosine receptor activates cAMP and calcium signaling // Insect Biochemistry and Molecular Biology. — 2007.— **37**, N 4.— P. 318-329.

80. *Tseng A.-S. K. and Hariharan I. K.* An Overexpression Screen in Drosophila for Genes That Restrict Growth or Cell-Cycle Progression in the Developing Eye // Genetics. — 2002.— **162**, N 1.— P. 229-243.

81. *Wolfgang W. J., Roberts I. J. H., Quan F., O’Kane C., and Forte M.* Activation of protein kinase A-independent pathways by Gsα in Drosophila // Proceedings of the National Academy of Sciences. — 1996.— **93**, N 25.— P. 14542-14547.

82. *Sturtevant M. A., Roark M., and Bier E.* The Drosophila rhomboid gene mediates the localized formation of wing veins and interacts genetically with components of the EGF-R signaling pathway // Genes Dev. — 1993.— **7**, N 6.— P. 961-973.

83. *Urban S., Lee J. R., and Freeman M.* A family of Rhomboiproteases activates all Drosod intramembrane phila membrane-tethered EGF ligands // The EMBO journal. — 2002.— **21**, N 16.— P. 4277-4286.

84. *Wasserman J. D., Urban S., and Freeman M.* A family of rhomboid-like genes: Drosophila rhomboid-1 and roughoid/rhomboid-3 cooperate to activate EGF receptor signaling // Genes Dev. — 2000.— **14**, N 13.— P. 1651-1663.

85. *Meyer F. and Aberle H.* At the next stop sign turn right: the metalloprotease Tolloid-related 1 controls defasciculation of motor axons in Drosophila // Development. — 2006.— **133**, N 20.— P. 4035-4044.

86. *Palmer R. H., Fessler L. I., Edeen P. T., Madigan S. J., McKeown M., and Hunter T.* DFak56 Is a Novel Drosophila melanogaster Focal Adhesion Kinase // J. Biol. Chem. — 1999.— **274**, N 50.— P. 35621-35629.

87. *Fischer S., Bayersdorfer F., Harant E., Reng R., Arndt S., Bosserhoff A.-K., and Schneuwly S.* fussel (fuss) - A Negative Regulator of BMP Signaling in Drosophila melanogaster // PloS one. — 2012.— **7**, N 8.—

88. *Baker S. E., Lorenzen J. A., Miller S. W., Bunch T. A., Jannuzi A. L., Ginsberg M. H., Perkins L. A., and Brower D. L.* Genetic Interaction Between Integrins and moleskin, a Gene Encoding a Drosophila Homolog of Importin-7 // Genetics. — 2002.— **162**, N 1.— P. 285-296.

89. *Chen G.-C., Lee J. Y., Tang H.-W., Debnath J., Thomas S. M., and Settleman J.* Genetic interactions between Drosophila melanogaster Atg1 and paxillin reveal a role for paxillin in autophagosome formation // Autophagy. — 2008.— **4**, N 1.— P. 37-45.

90. *Domínguez-Giménez P., Brown N. H., and Martín-Bermudo M. D.* Integrin-ECM interactions regulate the changes in cell shape driving the morphogenesis of the Drosophila wing epithelium // Journal of Cell Science. — 2007.— **120**, N 6.— P. 1061-1071.

91. *Gluderer S., Brunner E., Germann M., Jovaisaite V., Li C., Rentsch C. A., Hafen E., and Stocker H.* Madm (Mlf1 adapter molecule) cooperates with Bunched A to promote growth in Drosophila // J Biol. — 2010.— **9**, N 1.—

92. *Staudt N., Fellert S., Chung H.-R., Jäckle H., and Vorbrüggen G.* Mutations of the Drosophila Zinc Finger-encoding Gene vielfältig Impair Mitotic Cell Divisions and Cause Improper Chromosome Segregation // Mol. Biol. Cell. — 2006.— **17**, N 5.— P. 2356-2365.

93. *Zeng Y. A. and Verheyen E. M.* Nemo is an inducible antagonist of Wingless signaling during Drosophila wing development // Development. — 2004.— **131**, N 12.— P. 2911-2920.

94. *Chen D.-Y., Li M.-Y., Wu S.-Y., Lin Y.-L., Tsai S.-P., Lai P.-L., Lin Y.-T., Kuo J.-C., Meng T.-C., and Chen G.-C.* The Bro1 domain-containing Myopic/HDPTP coordinates with Rab4 to regulate cell adhesion and migration // Journal of Cell Science. — 2012.— N

95. *Eid J.-P., Arias A. M., Robertson H., Hime G. R., and Dziadek M.* The Drosophila STIM1 orthologue, dSTIM, has roles in cell fate specification and tissue patterning // BMC Dev Biol. — 2008.— **8**, N

96. *Mouchel-Vielh E., Rougeot J., Decoville M., and Peronnet F.* The MAP kinase ERK and its scaffold protein MP1 interact with the chromatin regulator Corto during Drosophila wing tissue development // BMC Dev Biol. — 2011.— **11**, N

97. *Schnorrer F., Kalchhauser I., and Dickson B. J.* The Transmembrane Protein Kon-tiki Couples to Dgrip to Mediate Myotube Targeting in Drosophila // Developmental Cell. — 2007.— **12**, N 5.— P. 751-766.

98. *Kiger Jr J. A., Natzle J. E., Kimbrell D. A., Paddy M. R., Kleinhesselink K., and Green M. M.* Tissue remodeling during maturation of the Drosophila wing // Developmental Biology. — 2007.— **301**, N 1.— P. 178-191.

99. *Batut J., Schmierer B., Cao J., Raftery L. A., Hill C. S., and Howell M.* Two highly related regulatory subunits of PP2A exert opposite effects on TGF-{beta}/Activin/Nodal signalling // Development. — 2008.— **135**, N 17.— P. 2927-2937.

100. *Guichard A., Srinivasan S., Zimm G., and Bier E.* A screen for dominant mutations applied to components in the Drosophila EGF-R pathway // Proceedings of the National Academy of Sciences. — 2002.— **99**, N 6.— P. 3752-3757.

101. *Donaldson T., Wang S.-H., Jacobsen T. L., Schnepp B., Price J., and Simcox A.* Regulation of the Drosophila Epidermal Growth Factor-Ligand Vein Is Mediated by Multiple Domains // Genetics. — 2004.— **167**, N 2.— P. 687-698.

102. *Toba G., Ohsako T., Miyata N., Ohtsuka T., Seong K. H., and Aigaki T.* The gene search system. A method for efficient detection and rapid molecular identification of genes in Drosophila melanogaster // Genetics. — 1999.— **151**, N 2.— P. 725-737.

103. *Rosin D., Schejter E., Volk T., and Shilo B.-Z.* Apical accumulation of the Drosophila PDGF/VEGF receptor ligands provides a mechanism for triggering localized actin polymerization // Development. — 2004.— **131**, N 9.— P. 1939-1948.

104. *Merino C., Reynaud E., Vázquez M., and Zurita M.* DNA Repair and Transcriptional Effects of Mutations in TFIIH inDrosophila Development // Mol. Biol. Cell. — 2002.— **13**, N 9.— P. 3246-3256.

105. *Hsiao F. C., Williams A., Davies E. L., and Rebay I.* Eyes Absent Mediates Cross-Talk between Retinal Determination Genes and the Receptor Tyrosine Kinase Signaling Pathway // Developmental Cell. — 2001.— **1**, N 1.— P. 51-61.

106. *Quinn L. M., Dickins R. A., Coombe M., Hime G. R., Bowtell D. D. L., and Richardson H.* Drosophila Hfp negatively regulates dmyc and stg to inhibit cell proliferation // Development. — 2004.— **131**, N 6.— P. 1411-1423.

107. *Vereshchagina N., Bennett D., Szoor B., Kirchner J., Gross S., Vissi E., White-Cooper H., and Alphey L.* The Essential Role of PP1? in Drosophila Is to Regulate Nonmuscle Myosin // Mol. Biol. Cell. — 2004.— **15**, N 10.— P. 4395-4405.

108. *Jones W. M., Chao A. T., Zavortink M., Saint R., and Bejsovec A.* Cytokinesis proteins Tum and Pav have a nuclear role in Wnt regulation // Journal of Cell Science. — 2010.— **123**, N 13.— P. 2179-2189.

109. *Ayyub C., Sen A., Gonsalves F., Badrinath K., Bhandari P., Shashidhara L. s., Krishna S., and Rodrigues V.* Cullin-5 plays multiple roles in cell fate specification and synapse formation during Drosophila development // Developmental Dynamics. — 2005.— **232**, N 3.— P. 865-875.
